# Recombinant production, purification, and biochemical characterization of a novel L-lactate dehydrogenase from *Bacillus cereus* NRC1 and inhibition study of mangiferin

**DOI:** 10.3389/fbioe.2023.1165465

**Published:** 2023-04-06

**Authors:** Sayed S. Esa, Ahmed F. El-Sayed, Mohamed I. El-Khonezy, Shubing Zhang

**Affiliations:** ^1^ Department of Cell Biology, School of Life Sciences, Central South University, Changsha, Hunan, China; ^2^ Molecular Biology Department, Biotechnology Research Institute, National Research Centre, Giza, Egypt; ^3^ Microbial Genetics Department, Biotechnology Research Institute, National Research Centre, Dokki, Giza, Egypt; ^4^ Egypt Center for Research and Regenerative Medicine (ECRRM), Cairo, Egypt

**Keywords:** lactate dehydrogenase, characterization, *Bacillus cereus*, molecular docking, cloning, mangiferin

## Abstract

Lactate dehydrogenase (LDH, EC 1.1.1.27) is one of the vital glycolytic conditions, especially during anaerobic conditions. It is a significant diagnostic, prognostic, and monitoring biomarker parameter. A 950-bp DNA fragment containing the gene (LDH) encoding LDH was amplified from *Bacillus cereus* NRC1. The deduced amino acid sequence reveals that *B. cereus* LDH (*Bc*-LDH) is highly homologous to the LDHs of *Bacillus* organisms. All LDH enzymes have a significant degree of conservation in their active site and several additional domains with unidentified functions. The gene for LDH, which catalyzes lactate synthesis, was cloned, sequenced (accession number: LC706200.1), and expressed in *Escherichia coli* BL21 (DE3). In this investigation, *Bc*-LDH was purified to homogeneity with a specific activity of 22.7 units/mg protein and a molecular weight of 35 kDa. It works optimally at pH 8.0. The purified enzyme was inhibited by FeCl_2_, CuCl_2_, ZnCl_2_, and NiCl, whereas CoCl_2_ was found to boost the activity of *Bc*-LDH. The molecular docking of the 3D model of the *Bc*-LDH structure with a natural inhibitor, mangiferin, demonstrated excellent LDH inhibition, with a free binding energy of −10.2 kcal/mol. Moreover, mangiferin is a potent *Bc*-LDH inhibitor that inhibits *Bc*-LDH competitively and has one binding site with a Ki value of 0.075 mM. The LDH-mangiferin interaction exhibits a low RMSF value (>1.5 Å), indicating a stable contact at the residues. This study will pave the way for more studies to improve the understanding of mangiferin, which could be considered an intriguing candidate for creating novel and improved LDH inhibitors.

## 1 Introduction

Lactate dehydrogenase (LDH, EC 1.1.1.27) is the final enzyme in glycolysis, which promotes the interconversion of pyruvate into lactate, which is pervasively prevalent in living organisms (Andreeßen et al., 2018; Naik et al., 2021). Liver diseases, malignancies, anemia, bone fractures, muscle damage, heart attack, infections like encephalitis and meningitis, HIV, and cancer are all correlated with elevated glycolysis and LDH activity (Nadeem et al., 2018; Farhana and Lappin, 2021). Additionally, the enzyme LDH is an attractive pharmacological target because it is among the potential biomarkers for predicting patients’ prognoses in COVID-19 (Kermali et al., 2020; Martinez Mesa et al., 2021). Increases in lactate production have beneficial effects on many processes, such as angiogenesis (Végran et al., 2011; De Saedeleer et al., 2012), invasiveness (Colen et al., 2011; Izumi et al., 2011), commensalism, inflammation (Colegio et al., 2014), and redox homeostasis (Doherty and Cleveland, 2013; Keddie et al., 2020). Furthermore, a metabolic symbiosis is established between glycolytic cancer cells that quickly generate lactate from glucose and oxidative cancer cells that prefer lactate-effective fuel source instead of glucose, which is called lactate metabolism (Sonveaux et al., 2008; Chiarugi et al., 2012). Lysosomal acidification and autophagy are further encouraged by the conversion of lactate to pyruvate by LDHs. It facilitates glycolysis and the regeneration of NAD^+^, representing promising targets in various diseases and cancer therapies (Ippolito et al., 2019).

*Bacillus cereus* is a gram-positive, spore-forming bacterium commonly found in the soil, dust, and various foods, such as grains, vegetables, and dairy products. While generally considered a foodborne pathogen, *B. cereus* is also recognized as an important industrial microorganism for its ability to produce various enzymes, antibiotics, and other biologically active compounds (Vidic et al., 2020).

In biomedical investigation, in order to construct calibration curves for enzyme activity assays, kinetic and stability investigations, and structural analysis, the pure enzyme may be desired (McDonald and Tipton, 2022). Several approaches, such as anion-exchange chromatography, cation-exchange chromatography, and gel filtration chromatography, have been employed to purify LDH and identify its isoenzymes (El-Sayed, 2011). The well-conserved tertiary structure of the LDH enzyme represents a dynamic active site crucial for controlling the ligand binding selectivity (Kayamba et al., 2021). Research findings on the conserved structural and functional domains of LDH from many species have led to the discovery of novel inhibitory compounds (Granchi et al., 2010); even though direct blocking of the LDH active site is problematic from a physicochemical and selectivity standpoint, such target site inhibition by some bioactive substances is urgent (Banu et al., 1992; Li et al., 2021; Thabault et al., 2021).

Typically, molecular docking aims to determine the ligand shape bound to the target with the most advantageous binding energy. Due to the flexibility of the ligand and protein, this is a difficult task. Two distinct conformational states of LDH5, a closed and an open conformation, have been observed concerning protein flexibility (Granchi et al., 2010). In addition, various ligands can interact with one another no matter whether the NADH co-factor is present or not. In light of this, a novel hLDH5 inhibitor could be discovered using an ensemble *vs.* docking method. An evaluation of the consensus docking approach was presented by Tuccinardi et al. (2014).

The naturally occurring bioactive polyphenolic compound, mangiferin (2-β-D-glucopyranosyl-1,3,6,7-tetrahydroxy-9H-xanthen-9-one) (Supplementary Figure S1) is an important potential curative agent against lifestyle-related disorders. It is found in many fruits and vegetables with a wide range of biological and pharmacological properties (Prabhu et al., 2006; Liao et al., 2011; Ochocka et al., 2017; Aziz et al., 2018; Reyes-Farias and Carrasco-Pozo, 2019). Mangiferin is a natural compound that is generally considered safe, with no reported adverse effects in humans. Many studies on mangiferin have been conducted in animal models (Lum et al., 2021; Dutta et al., 2023). The studies presented here seek to explain the direct inhibitory effect of the naturally occurring plant-based bioactive compound—mangiferin—on LDH activity and provide a spotlight on the compound's potential medical applications, particularly in oncotherapy.

Our study demonstrates how *B. cereus* produces LDH. Additionally, the *B. cereus* LDH gene for lactate dehydrogenase was amplified, cloned, sequenced, expressed, purified, and characterized. Additionally, a molecular docking study with mangiferin and a 3D *in silico* model of the recombinant enzyme is reported.

## 2 Material and methods

### 2.1 Chemicals, enzymes, plasmids, and bacterial strains

The chemicals, enzymes, plasmids, and bacterial strains were obtained from various sources. Sigma was the source for sodium pyruvate, NADH, lithium lactate, NAD^+^, nitroblue tetrazolium salt (NBT), Coomassie brilliant blue G, Sephacryl S-300, and DEAE-cellulose, as well as mangiferin. Thermo Fisher Scientific provided the remaining chemical reagents of analytical grade, which included Taq DNA polymerase, dNTPs, T4 DNA ligase, the InsTAclone PCR Cloning Kit, DNA and protein molecular weight markers, restriction endonucleases, and the GeneJET Genomic DNA Purification and Gel Extraction Kits. Macrogen Inc. was the source for oligonucleotide primers. The *Escherichia coli* strains DH5α™ and BL21-CodonPlus (DE3)-RIL were obtained from Stratagene, while Novagen provided the expression vector [pET-28a (+)].

### 2.2 Screening and selection of LDH-producing bacterial isolate

Different kinds of bacteria were obtained from the local isolate to screen for the LDH enzyme from Cairo, Egypt. For the isolation of microorganisms, 1 g of various soil samples were obtained from the Al Qalyubia Governorate in Egypt and introduced into fresh 100 ml salt medium [(g/l): glucose, 10; NaNO_3_, 0.5; KCl, 0.5; MgSO_4_.7H_2_O, 0.5; K_2_HPO_4_, 1.0; FeSO_4_.7H_2_O, 0.001] and incubated at 37°C for 48 h (El-Sayed et al., 2019).

### 2.3 Molecular identification of most promising isolate by 16s rRNA PCR and phylogenetic analysis

The extraction of the genomic DNA was carried out using the GeneJET Genomic DNA Purification Kit from Thermo Fisher Scientific in Lithuania, according to the manufacturer’s instructions. The PCR amplification that was performed using the forward primer 8F and reverse primer 1492R is listed in Table 1 The PCR mixture consisted of 50 μl, which included 22 μl of MQ, 25 μl of DreamTaq Green DNA Polymerase from Thermo Fisher Scientific in the United States, 1 μl each of the 10 μmol/l forward and reverse primers (synthesized by IDT), and 1 μl of the template. The PCR amplification protocol involved a preheating stage of 4 min at 95°C, followed by 30 s of denaturation at 95°C, 45 s of primer annealing at 50°C, 1 min of extension at 72°C, and a final extension step of 10 min at 72°C, and this cycle was repeated 35 times. A thermal cycler was utilized for the reactions (Applied Biosystems thermal cycler, Thermo Fisher Scientific, United States). Macrogen in Korea purified and sequenced the PCR products. The sequencing data were adjusted using the FinchTV version 1.4.0 tool. The BLASTN tool was applied to conduct an analysis of 16s rRNA sequences. The ClustalW 2.1 tool was applied for multiple sequence alignment. MEGAX implemented the neighbor-joining method to create the phylogenetic trees.

**TABLE 1:** Screening of lactate dehydrogenase from locally isolated bacteria.

Sample	Activity (U/ml)	Protein content (mg/ml)	Specific activity (U/mg/ml)
NRC1	3.2	2.9	1.1
NRC2	2.4	3	0.8
NRC3	1.5	2	0.75
NRC4	1.1	1.5	0.73
NRC5	0.6	1.6	0.37
NRC6	0.7	3	0.23
NRC7	0.4	1.7	0.23
NRC8	0.5	2.5	0.2
NRC9	0.3	2	0.15
NRC10	0.07	2	0.03
NRC11	0.05	1.8	0.02

### 2.4 Molecular cloning, sequencing, and expression of the LDH gene from *B. cereus*

#### 2.4.1 PCR amplification of the LDH gene from *B. cereus*

The LDH gene from *B. cereus* (*Bc*-LDH) was amplified through the polymerase chain reaction (PCR) technique, which uses the primer sequences specified in Table 1. The primer sequences were 5′-GGA​TCC​ATG​AAA​AAA​GGT​ATC​AAT​CGT​GT-3′ and 5′-AAG​CTT​TTA​TAG​TAC​TGG​TGC​CAT​TGT​T-3′, which were designed to contain restriction sites for the enzymes BamHI and HindIII. This resulted in the formation of sticky ends along the entire open reading frame (ORF) of the *Bc*-LDH gene. The PCR technique had a total volume of 25 μl, composed of different components such as 0.75 mM dNTPs, Taq polymerase buffer containing KCl, MgCl_2_, and Taq polymerase, 20 p.m. of each primer, and 2 μl of the diluted DNA template. The reaction mixture underwent thermal cycling, starting with a denaturation phase at 94°C for 4 min, followed by another denaturation phase of 30 s at 94°C, annealing at 52°C for 1 min, and extension at 72°C for 1.5 min. This cycle was repeated 35 times, and the final extension was performed at 72°C for 25 min to add a poly-A sequence to the end of the amplified fragment.

#### 2.4.2 Gene cloning and expression

The *Bc*-LDH gene was purified and then ligated into the pTZ57R/T plasmid to be used for transforming *E. coli* DH5α™ cells. Blue–white screening was used to identify colonies that had been effectively transformed with recombinant plasmids, and the results were validated by the sequencing analysis of isolated pTZ57R/T-*Bc*-LDH. *Hin*dIII and *Bam*HI were used to cleave the *Bc*-LDH gene from the pTZ57R/T-*Bc*-LDH vector, and then the *Bc*-LDH gene was ligated into the pET28a(+) vector. The procedure outlined in the InsTAclone Kit by Thermo Fisher Inc. was followed to perform the transformation of BL21 (DE3) cells using the ligation mixture. A single colony of the BL21 cells with the desired gene was selected and grown overnight in LB broth with the addition of kanamycin at a concentration of 50 μg/ml. A 1% sample of the overnight culture was utilized for further bacterial growth, which was incubated at 37°C with continuous agitation at 150 rpm. The culture was then stimulated for gene expression by adding 0.1 mM IPTG and was allowed to grow at 37°C for 24 h.

#### 2.4.3 Bioinformatics and sequence analysis of LDH gene

The recombinant *Bc*-LDH gene was sequenced by Macrogen, Korea, and the sequence analysis was accomplished by BLAST. ClustalW was used to perform multiple sequence alignments, and SignalP examined the signal peptide. ExPA Sy made predictions about the isoelectric point (pI) and molecular weight. A Conserved Domain Search was used to investigate the conserved domains of the deduced amino acid sequence. The MEGA11 software was used that applied the neighbor-joining method to construct a phylogenetic tree (Tamura et al., 2021).

#### 2.4.4 Nucleotide sequence accession number

16s rRNA gene sequence of *B. cereus* strain NRC1 was submitted to the GenBank database with accession number: ON231812. Also, the *Bc*-LDH gene was sequenced and uploaded to the GenBank (accession number: LC706200.1).

### 2.5 Enzyme assay and protein determination

#### 2.5.1 Lactate dehydrogenase assay

The measurement of the lactate dehydrogenase (LDH) activity was performed using the method described by Nadeem et al. (2011). A reaction mixture of 1 ml was prepared, which consisted of 50 μl of the enzyme, 0.22 mM NADH, 0.2 mM sodium pyruvate, and 0.1 M phosphate buffer with a pH of 7.5. The absorbance was recorded at 340 nm. The activity of LDH was expressed in units, where one unit was defined as the amount of enzyme that caused a reduction in optical density by 1.0 per minute. The isoenzyme patterns of the LDH were examined on polyacrylamide gels by staining with a solution that contained 50 ml of 0.1 M Tris-HCl, pH 8.0, 288 mg of lithium lactate, 14 mg of NAD^+^, 3.5 mg of NBT, and small amounts of PMS. The gels were incubated at 37°C until the development of dark blue bands was observed (Whitt, 1970).

#### 2.5.2 Determination of protein concentration

The concentration of protein content was determined using the Bradford method (Bradford, 1976) with bovine serum albumin as the reference.

### 2.6 Purification and molecular weight of lactate dehydrogenase

#### 2.6.1 Purification

The process of purifying *Bc*-LDH involved several steps. First, a colony of BL21 cells was grown overnight in the LB broth medium containing kanamycin (50 μg/ml) and then collected by scraping and suspending in 0.1 M phosphate buffer (pH 7.5). The cells were centrifuged twice, and the pellet was treated with lysozyme and mechanically disrupted. The resulting mixture was then recentrifuged, and the supernatant was treated with solid ammonium sulfate twice to reach a saturation concentration of 30 and 80%, respectively. The precipitate was dialyzed overnight using 0.1 M phosphate buffer (pH 7.5), centrifuged, and then poured onto a DEAE-cellulose column. The LDH activity fractions were collected after elution using a stepwise gradient of NaCl in phosphate buffer (pH 7.5). The high LDH activity fractions were then loaded onto a CM-cellulose column and eluted using a linear gradient of NaCl in sodium acetate buffer (pH 5.5). Finally, the LDH activity fractions were loaded onto a Sephacryl S-300 column and eluted using 0.02 M phosphate buffer (pH 7.5).

#### 2.6.2 Molecular weight determination

The molecular weight of the enzyme was measured by gel filtration on a Sephacryl S-300 column (142 cm × 1.75 cm i.d.). The column was calibrated using a range of protein standards, such as *ß*-amylase (200,000), alcohol dehydrogenase (150,000), bovine serum albumin (66,000), carbonic anhydrase (29,000), and cytochrome C (12,400). Dextran blue (2,000,000) was used to determine the void volume, according to the method described by Andrews (1964). The subunit molecular weight was determined using sodium dodecyl sulphate–polyacrylamide gel electrophoresis (SDS-PAGE) (Laemmli, 1970), and calibration was done by using a standard pre-stained protein marker (BlueStar PLUS).

### 2.7 Characterization of lactate dehydrogenase

The optimal pH range for *Bc*-LDH was determined by measuring its activity in 20 mM buffers of sodium citrate with a pH range of 3.0–4.5, sodium acetate with a pH range of 4.5–6.0, sodium phosphate with a pH range of 6.5–8.0, Tris-HCl with a pH range of 8.0–9.0, and glycine-NaOH with a pH range of 9.0–11.0. The residual activity of the enzyme was also assessed by pre-incubating it at 37°C for 30 min with different metal ions at 2 and 5 mM concentrations.

### 2.8 *In silico* studies of LDH from *B. cereus*

#### 2.8.1 Secondary structure prediction

The primary LDH protein sequences of the *B. cereus* strain NRC were collected from the NCBI protein sequence database. BLASaTp was used to identify the template structure in the Protein Data Bank (PDB) database that was most similar to the LDH model. The LDH sequence was used as a search query. Then, the sequences of the target and template were aligned using the Clustal Omega algorithm with default parameters. The DSSP program was then used to predict the secondary structure (Eswar et al., 2006).

#### 2.8.2 Homology modeling and model validation

The creation of a 3D model of LDH using homology modeling techniques was carried out using Modeller v9.20 (Eswar et al., 2006). Then, 3D models were constructed and graded according to the Discrete Optimized Protein Energy (DOPE) scores provided by the Modeller software. Using the Protein Preparation Wizard and a force field, the energy of the constructed models was minimized.

### 2.9 Molecular docking of natural inhibitors on lactate dehydrogenase

#### 2.9.1 Lactate dehydrogenase receptor preparation

The Modeller was used for producing 3D models of the LDH protein with multiple PDB identification codes (Webb and Sali, 2016). After that, using the MGL Tools of AutoDock Vina (Eberhardt et al., 2021), hydrogen atoms were incorporated into the receptor molecule. The target protein was subsequently saved as a dockable PDBQT file for molecular docking.

#### 2.9.2 Preparation of inhibitor ligand

Mangiferin with SDF format was retrieved from PubChem, https://pubchem.ncbi.nlm.nih.gov/. Using the Open Babel, each inhibitor was transformed to mol2 (O’Boyle et al., 2011). Additionally, the polar hydrogen charges were assigned using the Gasteiger method, and the internal degrees of freedom and torsions were optimized to be at their minimum values. The AutoDock tools were then utilized to convert the ligand molecules to the dockable PDBQT format.

#### 2.9.3 Docking studies

The purpose of the docking studies was to investigate the binding mode of the proposed 3D model of LDH protein using AutoDock tools 4.2. Before docking, the AutoGrid program created ligand-centered maps with 0.375 A spacing and a grid size of 90 A × 90 A × 90 A. The binding interactions between the ligands and three-dimensional model of the LDH protein were visualized using the PyMOL program. All the docking experiments were performed using AutoDock Vina because it offers (a) more accuracy in predicting ligand–protein interaction when compared to its previous AutoDock 4.2; (b) shorter running time; and (c) more accuracy for ligand processing, more than 20 rotatable bonds. With AutoDock Vina, all the docking experiments were done using the blind docking method.

#### 2.9.4 Visualization

The analysis of the 2D hydrophobic bonds, hydrogen bonds, their bond lengths, and hydrogen-bond interactions of the amino acid of the receptor with a ligand was utilized using the BIOVIA Discovery Studio 4.5 program (BIOVIA, Dassault Systèmes, 2021).

#### 2.9.5 Molecular dynamics simulation

The binding interactions and binding affinities of the protein–ligand complexes were examined through the use of molecular dynamics (MD) simulation. The MD simulation was performed using the Desmond simulation software of Schrödinger LLC (Schrödinger Release, 2021). The NPT ensemble for a temperature value of 300 K and pressure of 1 bar was applied in all runs. The simulation time used was 100 ns with a relaxation time of 1 ps. In all simulations, we used the force field parameters OPLS_2005. To control the stability of the MD simulations, we examined the root mean square deviation (RMSD), root mean square fluctuation (RMSF), and radius of gyration (rGyr) calculations of the protein and ligand atom positions over time.

### 2.10 Determination of Ki of mangiferin on lactate dehydrogenase

The effects of inhibition of mangiferin on *Bc*-LDH at concentrations ranging from 0.08 to 1.0 mM were examined to determine the inhibition constant (Ki). To evaluate how mangiferin affects the LDH activity, the Lineweaver–Burk and Dixon plots were created and used to obtain the Ki (Dixon and Webb, 1979).

### 2.11 Statistical analysis

Each experiment was carried out thrice in duplicates, and the standard deviations of the results were determined using Microsoft Excel. The data were considered as mean ± S.E. (*n* = 3).

## 3 Results

### 3.1 Isolation and screening for lactate dehydrogenase–producing bacteria

Eleven culturable bacteria were isolated from agricultural soil for the current study. They all acquired specific LDH activities between 0.02–1.1 U/mg/ml (Table 2). Because of its high production rate among these LDH-producing bacteria, an isolate NRC1 was chosen.

**TABLE 2:** Strains, plasmids, and primers used in this study.

Strains, plasmids, and primers		Characteristics	Source
Strains	*E. coli* DH5α™	Host strain for cloning vector pET-28a (+)	TaKaRa
	*E. coli* BL21 (DE3)	Host strain for expression vector pET-28a (+)	TaKaRa
	*E. coli* BL21-(LDH)	*E. coli* BL21 harboring LDH	This study
	*B. cereus* NRC1	Wild-type strain with LDH	This study
Plasmids	pTZ57R/T	Cloning vector	Invitrogen
	pTZ57R/T-*Bc*-LDH	Cloning vector carrying *Bc*-LDH	This study
	pET-28a(+)	Expression vector with T7 promoter, Kan^r^	Invitrogen
	pET28-*Bc*-LDH	pET-28a (+) carrying *Bc-*LDH	This study
Primer	LDH-F	5′-GGA​TCC **ATG** AAA​AAA​GGT​ATC​AAT​CGT​GT-3′	This study
	LDH-R	5′-AAG​CTT **TTA** TAG​TAC​TGG​TGC​CAT​TGT​T-3′	
	8 F	5′-AGA​GTT​TGA​TCC​TGG​CTC​AG-3′	Universal
	1492R	5-GGT​TAC​CTT​GTT​ACG​ACT​T-3′	

### 3.2 Identification of selected lactate dehydrogenase–producing bacteria

**FIGURE 1:**
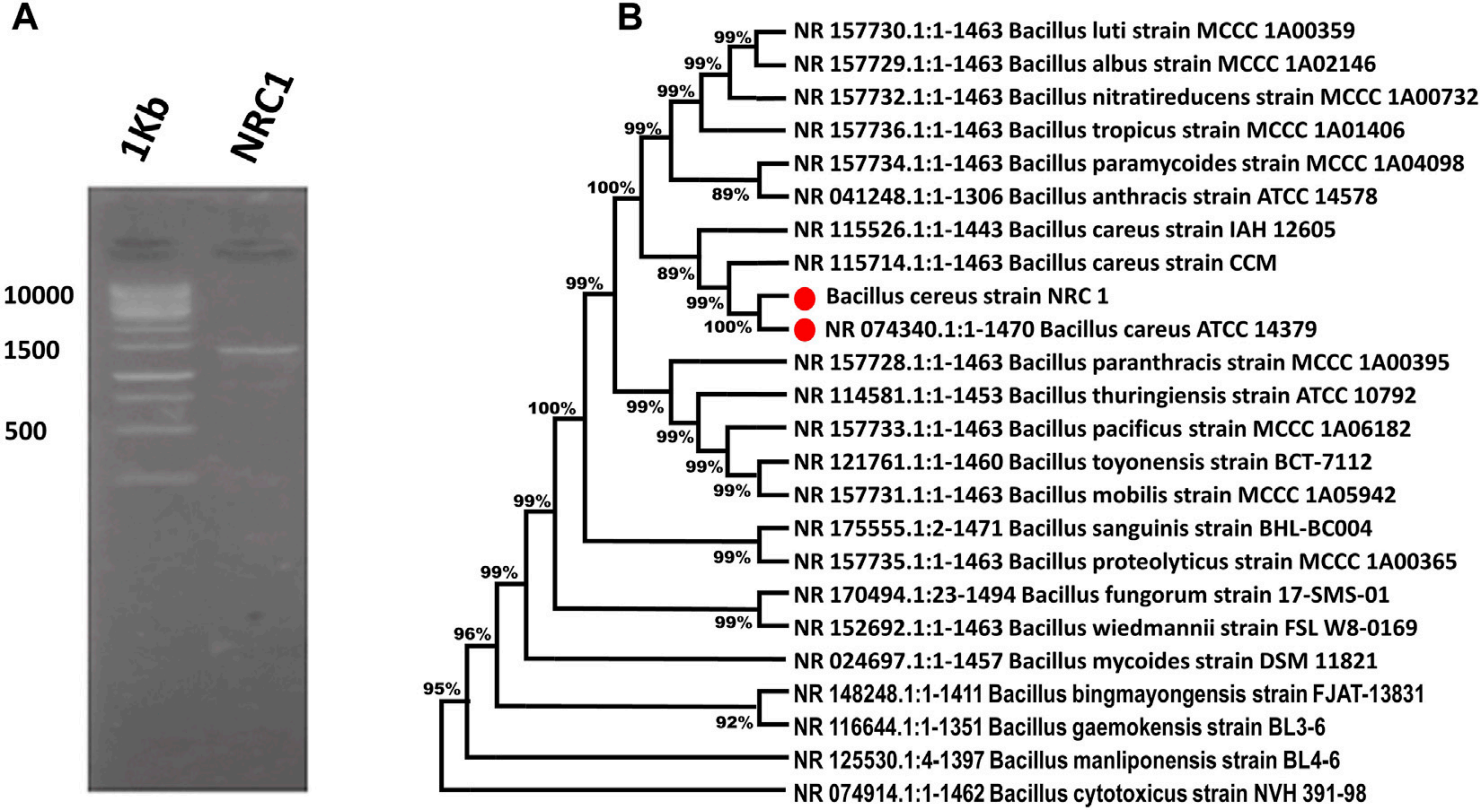
**(A)** Bacterial identification PCR—Identified by 16S rRNA Sequences. **(B)** Phylogenetic analysis produced as a result of the repeated alignment of the 16s rRNA gene sequence of *Bacillus cereus* NRC1 with the sequences of other *Bacillus* strains located in the GenBank database.

This study describes the identification and taxonomy of an isolate NRC1 isolated from soil. The bacterium was recognized as a *Bacillus* species and named *Bacillus cereus* NRC1. It was more closely related to *B. cereus* in taxonomy according to its 16s rRNA sequencing gene 1500 bp as presented in Figure 1A. Based on an initial assessment of the isolated *Bacillus* sp., the most active strain was chosen. The 16s rRNA gene sequence was obtained, located, and aligned against other sequences discovered and made accessible in the GenBank database using the BLAST tool. This allowed researchers to determine the similarity score and calculate the statistical significance of the matches. The findings of the phylogenetic tree showed that this bacterium was taxonomically closer to *B. cereus*, and the isolate NRC1 shared 99.04 percent homology with *B. cereus* ATCC and 99.33% with *B. cereus* strain CCM 201 (Figure 1B). The strain NRC1 was identified as *B. cereus* NRC1 based on an examination of its DNA sequence, and it was submitted to GenBank with the accession number ON231812.

### 3.3 Complete sequencing, cloning, and isolation of the *B. cereus* LDH gene

**FIGURE 2:**
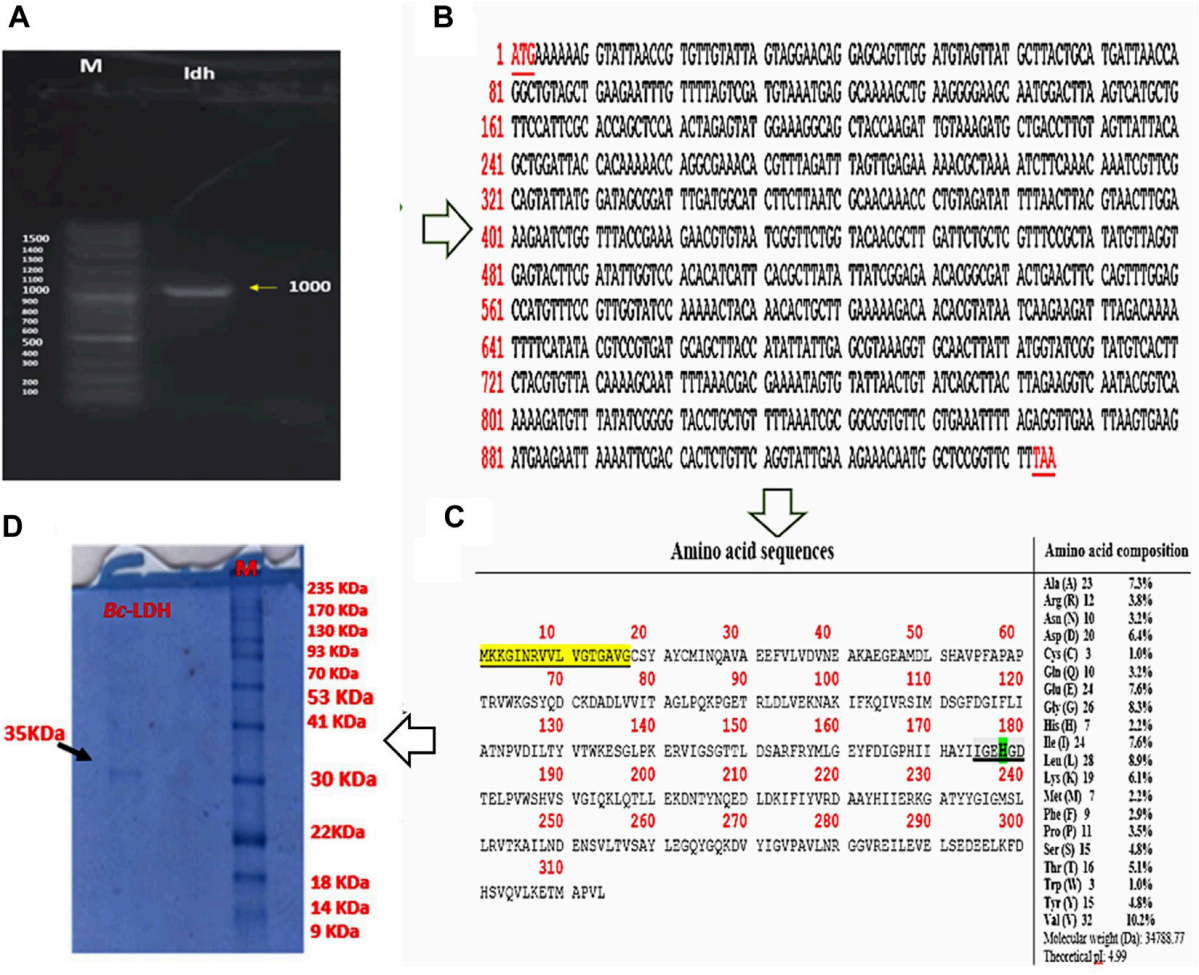
**(A)** PCR amplification of 0.95 kb *Bc*-LDH gene from the chromosomal DNA of *Bacillus cereus* NRC1. **(B)** Complete nucleotide sequence of the *Bc*-LDH gene from *B. cereus* open reading frame (ORF) nucleotide sequence. **(C)** Complete amino acid sequences and composition of the *Bc*-LDH gene from *B. cereus* NRC1. **(D)** SDS-PAGE showing a single band with a molecular weight of 35 kDa.

The study demonstrates the potential of using novel strains of *B. cereus* NRC1 to amplify and clone the *Bc*-LDH gene, which could have significant applications in producing industrially important products. Using novel strains of *B. cereus* NRC1, *Bc*-LDH gene’s 945-bp gene length was amplified using PCR and cloned with the pTZ57R/T plasmid to generate the construct pTZ57R/T-*Bc*-LDH (Figure 2A and Supplementary Figure S2A). By using colony PCR and the extracted plasmid sequencing analysis, the transformed cells were verified. The ORF contained a full-length *Bc*-LDH gene, which codes for a 314 amino acid protein. The *Bc-*LDH gene was sequenced and uploaded to GenBank (accession number LC706200.1) (Figures 2B,C). Also, the *Bc*-LDH gene from the pTZ57R/T-*Bc*-LDH construct was cleaved using the restriction enzymes *Bam*HI and *Hin*dIII, which resulted in the creation of the recombinant plasmid pET28-*Bc*-LDH. The plasmid pET28-*Bc*-LDH was used to transform the BL21 (DE3) strain of *E. coli* into competent cells (Supplementary Figure S2B). In addition, the SDS-PAGE homogeneity assessment revealed that the purified *Bc*-LDH enzyme moved in a single band with a molecular weight of 35 kDa (Figure 2F).

### 3.4 Bioinformatics and sequence analysis of L-lactate dehydrogenase protein

**FIGURE 3:**
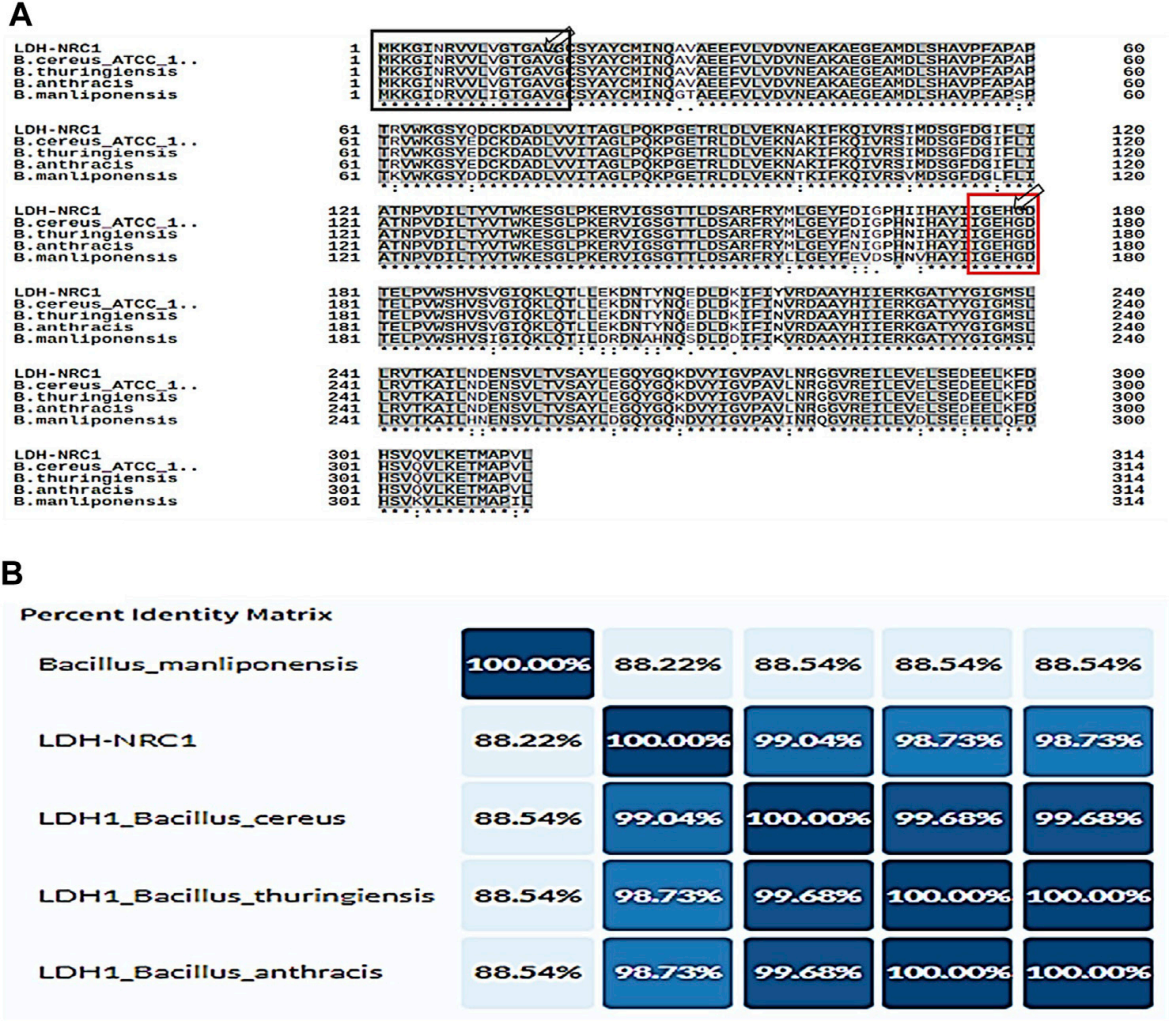
**(A)** LDH gene sequences from *Bacillus cereus* NRC1 and other organisms aligned based on their recognized amino acid sequences. The following is a list of accession numbers for the other sequences: LDH-NRC1, *B. cereus* ATCC, *Bacillus thuringiensis*, *Bacillus anthracis*, and *Bacillus manliponensis* LDH, whose GenBank accession numbers are BDI00612.1, Q81EP4, Q6HK31, Q81RW4, and A0A073K0E2, respectively. Identity is indicated by a “*” symbol and similarity is indicated by a “**:**” symbol. Also, the red box shows active site positions and the black box represents the signal peptide region (1–17 residues). **(B)** Percent identity matrix of LDH protein of *B. cereus* NRC1 and other organisms.

The study aims to investigate the amino acid sequence, function, and phylogenetic analysis of the *Bc*-LDH enzyme. The amino acid sequence of *Bc*-LDH was inferred from its nucleotide sequence and then multiple sequences aligned with four additional L-lactate dehydrogenases from the reviewed protein database, UniProt. *Bc*-LDH had a 99.04% similarity with the *B. cereus* ATCC strain with accession number Q81EP4 and 98.73% with both *Bacillus thuringiensis* and *Bacillus anthracis* with accession numbers Q6HK31 and Q81RW4, respectively (Figure 3A). The percent identity matrix of the *Bc*-LDH protein and other L-LDH proteins is illustrated in Figure 3B.

The prediction of gene function based on the analysis of the conserved domain of the *Bc*-LDH gene was performed using the Conserved Domain Database (CDD) of NCBI. The result showed that amino acid residues ranging from 1 to 313 belonged to the L-LDH protein family domain. Subsequently, the composition of amino acids and their percentages are depicted, and the *Bc*-LDH protein has a predicted molecular weight of 35.3 kDa and an isoelectric pH of 8.16 (Figure 2D). Furthermore, using the ScanProsite software, six amino acids made up the LDH’s active site, which is between amino acids 175 and 181. In addition, investigations identified one residue in the *Bc*-LDH active site (His178) that acted as the catalytic proton acceptor; represented as a red box in Figure 3A.

**FIGURE 4:**
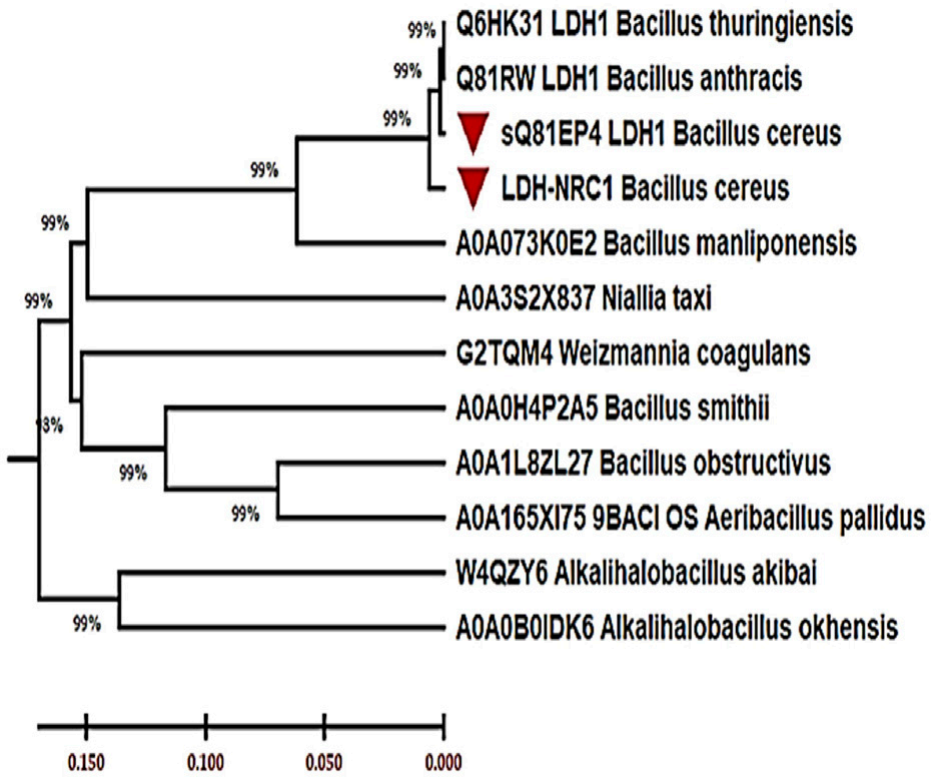
Phylogenetic tree of *Bc*-LDH proteins from *Bacillus cereus* strain NRC1 and other L-lactate dehydrogenase proteins inferred using the neighbor-joining method (MEGA X).

Also, the SignalP 6.0 server assessed the signal peptides and their cleavage sites, and the results covered residues ranging from 1 to 17 (Supplementary Figure S3). To locate *Bc*-LDH among the other members of the recognized LDH family, the phylogenetic analysis was next carried out (Figure 4). Each of the 11 curated L-LDH proteins that were found in the reviewed UniProt protein database was linked to different types of organisms, and they were all chosen for the phylogenetic analysis. The data set included LDH proteins from bacteria that showed 99% sequence similarity to comparable bacterial L-LDH proteins, according to the comparative analyses (e.g., *B. cereus*). The results provided valuable insights into the properties and function of the *Bc*-LDH enzyme, which could have implications for various biotechnological applications.

### 3.5 Purification of recombinant lactate dehydrogenase

**FIGURE 5:**
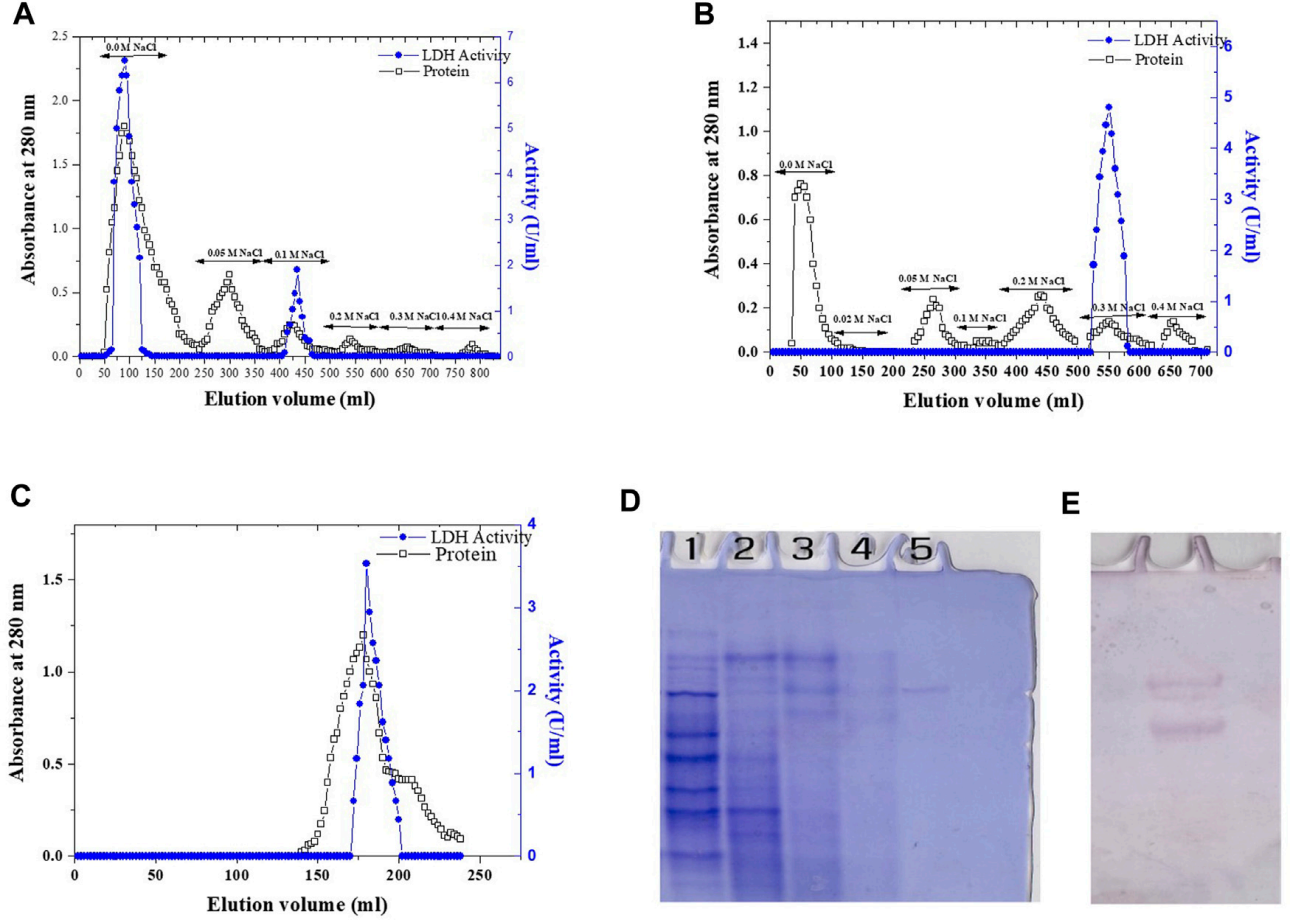
Elution profile of *Bc-*LDH: protein UV absorption and LDH activity. **(A)** Elution profile on DEAE-cellulose, pH 7.6. **(B)** Elution profile of *Bc-*LDH DEAE-cellulose fractions on CM-cellulose column, pH 5.5. **(C)** *Bc-*LDH CM-cellulose fractions on Sephacryl S-300 column, pH 7.6. **(D)** 7% native PAGE of LDH pattern. **(E)** LDH isoenzyme activity.

In this study, the purification process of recombinant *Bc*-LDH enzyme was carried out by using various chromatography techniques. The *E. coli* strain transfected with pET28a-*Bc*-LDH was cultured at a temperature of 37°C and induced by 0.1 mM IPTG for 24 h. Then, the total soluble proteins were extracted and separated by SDS-PAGE in order to distinguish the induced protein. The purification of the recombinant *Bc*-LDH is summarized in Table 3. Finely powdered ammonium sulfate was applied to the crude extract. The dialyzed ammonium sulfate–concentrated enzyme was fractionated on a DEAE-cellulose column. The majority of the *Bc*-LDH activity was extracted at the NaCl concentration of 0.0 M, while 0.1 M NaCl peaked with low level (Figure 5A). The high *Bc*-LDH fractions were applied on cation exchange chromatography CM-cellulose, where *Bc*-LDH activity was eluted at 0.3 NaCl (Figure 5B). Purified *Bc*-LDH retained 32% of its initial activity when fractions were eluted on a Sephacryl S-300 column, recording a specific activity of 22.7 units/mg protein and 25.5 purification fold (Figure 5C). The molecular weight estimated by using a Sephacryl S-300 gel filtration calibration curve is given as 149.5 kDa (Supplementary Figure S4). The electrophoretic profile of proteins during the SDS-PAGE homogeneity assessment revealed that the purified *Bc*-LDH enzyme moved in a single band with a molecular weight of 35 kDa (Figure 2F; Figures 5D, E).

**TABLE 3:** Typical purification scheme for LDH from *Bacillus cereus.*

Purification step	Protein (mg)	Specific activity (U/mg protein)	Fold purification	Recovery (%)
Crude extract				
	87	0.89	1	100
Ammonium sulfate ppt.	70.3	0.97	1.1	87.8
(30%–80%)				
DEAE-cellulose				
0.0 NaCl	10	5.1	5.7	65
0.1 NaCl	4	2.25	2.5	11.5
CM-cellulose				
0.3 NaCl	5	6.8	7.6	46
Sephacryl S-300				
	1.1	22.7	25.5	32

### 3.6 Characterization of purified lactate dehydrogenase

**FIGURE 6:**
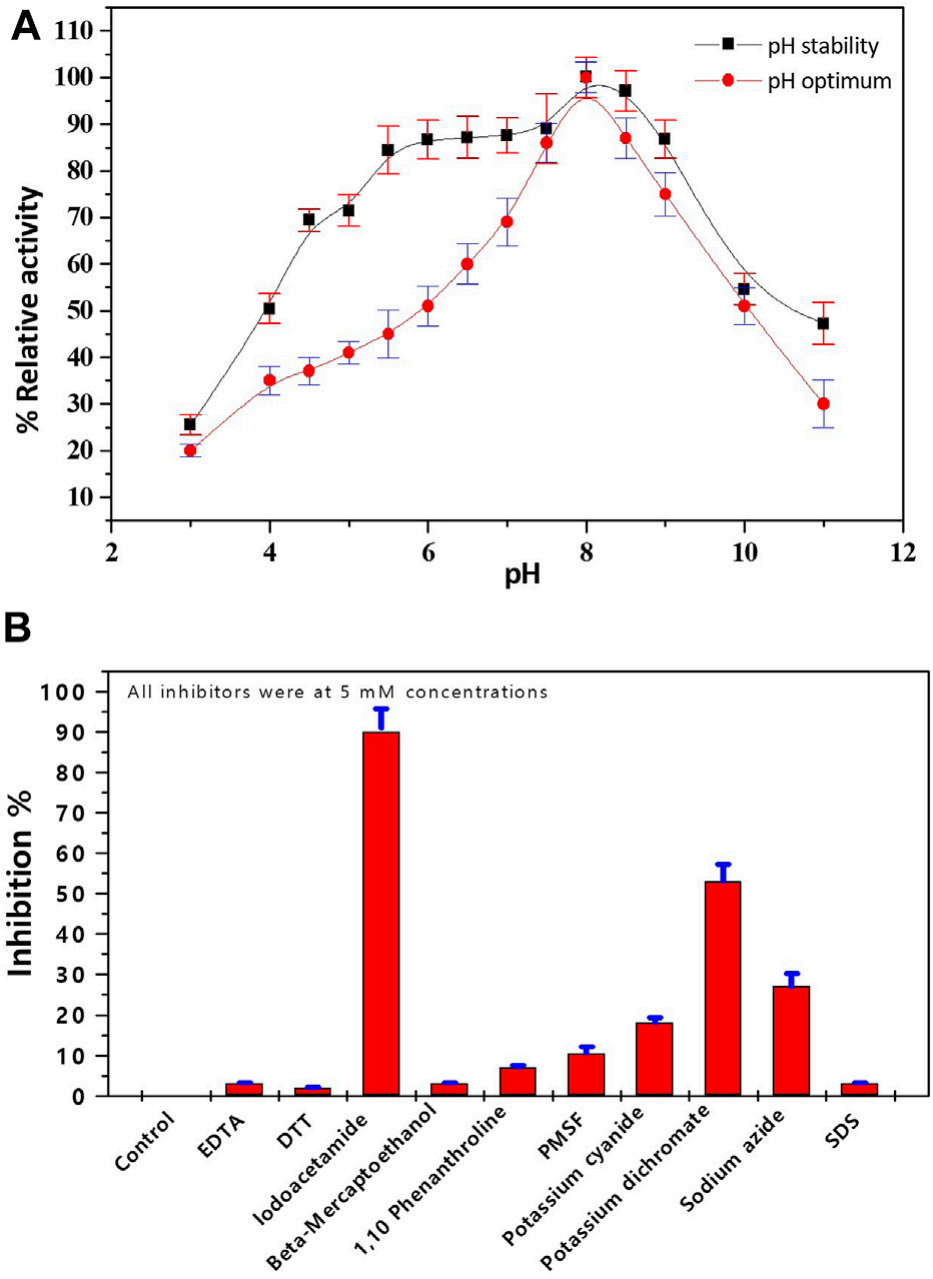
**(A)** Impact of pH on the purified LDH, using 0.05 M of pHs ranging from 3.0 to 11. **(B)** Effect of inhibitors on the purified LDH from *Bacillus cereus*.

The impact of pH and various divalent metal cations and inhibitors on the activity of pure *Bc*-LDH was investigated. At various pH values between 3.0 and 11.0, the impact of pH on pure *Bc*-LDH was evaluated. The ideal pH for *Bc*-LDH functioning was discovered to be pH 8.0. *Bc*-LDH retained most of its initial activity after being pre-incubated at pH ranging from 5.5 to 9.0 (Figure 6A). Table 1S displays how different divalent metal cations affect the activity of *Bc*-LDH. Most divalent cations, such as FeCl_2_, CuCl_2_, ZnCl_2_, MnCl_2_, CaCl_2_, and NiCl_2_, inhibited pure *Bc*-LDH, although CoCl_2_ and MgCl cations were activators for *Bc*-LDH even at 2 mM, and this effect was more prominent at 5 mM. The effect of various distinct and characteristic inhibitors on pure *Bc*-LDH is shown in the current study (Figure 6B). Figure 6B shows the percentage of *Bc*-LDH inhibition after pre-incubating with purified *Bc*-LDH at 37°C for 15 min. While EDTA, DTT, iodoacetamide, *ß*-mercaptoethanol, SDS, and PMSF showed negligible inhibition, potassium dichromate (K_2_Cr_2_O_7_) and sodium azide (NaN_3_) both inhibited *Bc*-LDH by 53% and 27%, respectively. The serine protease inhibitor PMSF did not inhibit pure *Bc*-LDH, indicating that these isoenzymes’ active sites were devoid of serine residues. It is possible that histidine residue has a crucial impact on the activity and structure of the enzyme because iodoacetamide exerted a significant influence on the pure *Bc*-LDH isoenzyme.

### 3.7 Secondary structure prediction of L-lactate dehydrogenase protein

**FIGURE 7:**
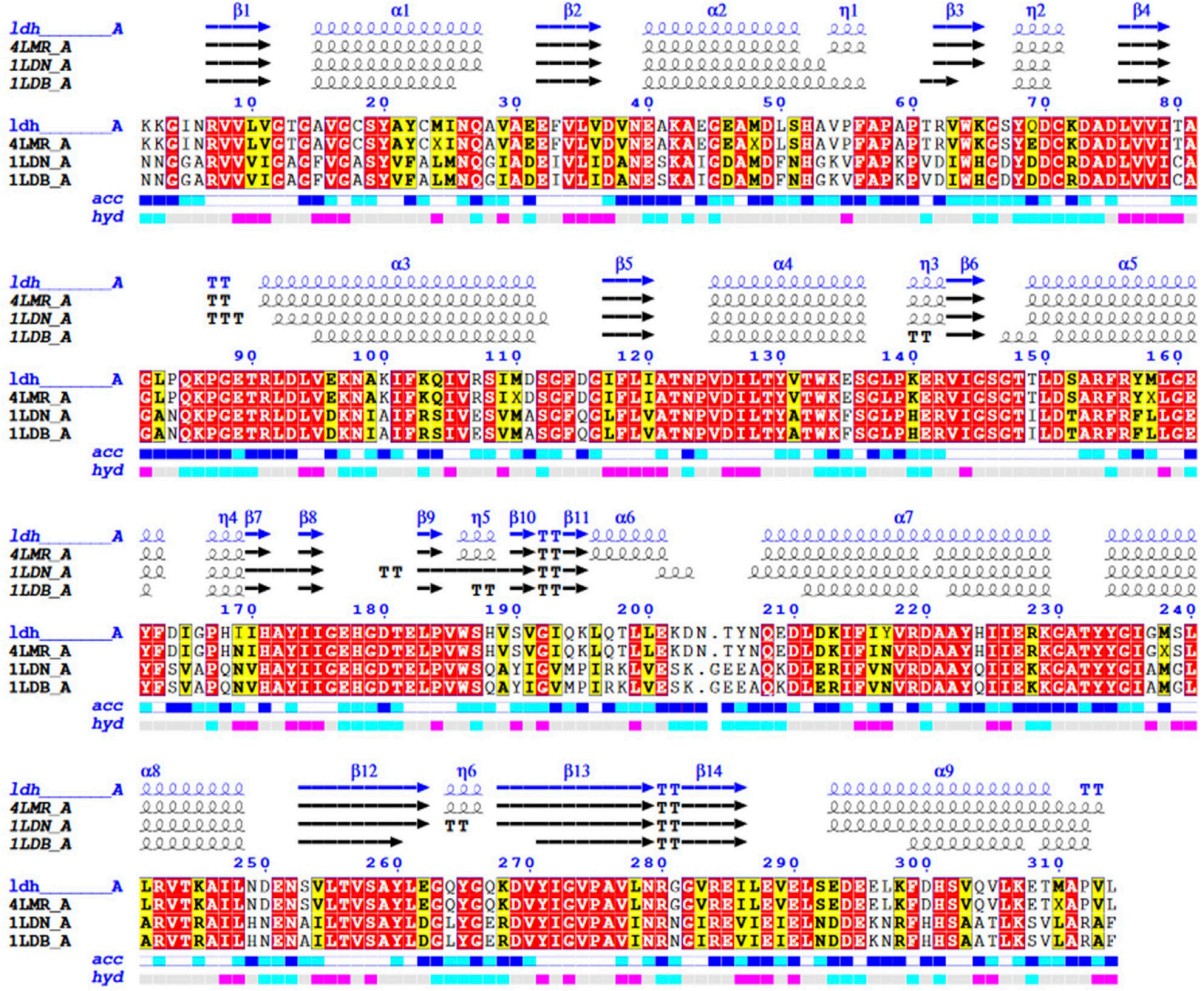
Structural alignment of the LDH gene from *Bacillus cereus* with *B. cereus* (PDB 4LMR_A). The actual secondary structure of the templates and the predicted secondary structure of the lactate dehydrogenase gene are shown above the alignment. The symbols η, α, and *ß*, respectively, stand for the secondary structure components 310 helices, *a*-helices, and *ß*-strands.

Our work was conducted to describe the amino acid sequence of *Bc*-LDH and its similarity to other proteins from *B. cereus* and *Bacillus stearothermophilus*. The amino acid sequence of *Bc*-LDH was submitted to the GenBank with the accession number BDI00612.1; this sequence consists of 314 amino acids. A protein sequence homology search was performed using the Protein-BLAST algorithm (BLASTp) to query the PDB database. It was found to share a high degree of similarity with proteins from *B. cereus* (PDB ID 4LMR_A; identity 97.12%) and *B. stearothermophilus* (PDB ID 5LDN A; identity 62.26%), both of which could serve as templates for comparative modeling. Figure 7 shows the secondary structure prediction of the *Bc*-LDH protein and structure alignment of LDH and templates. The *Bc*-LDH protein comprises nine *a*-helices and a *ß*-sheet (14 strands).

### 3.8 *In silico* homology modeling and validation

**FIGURE 8:**
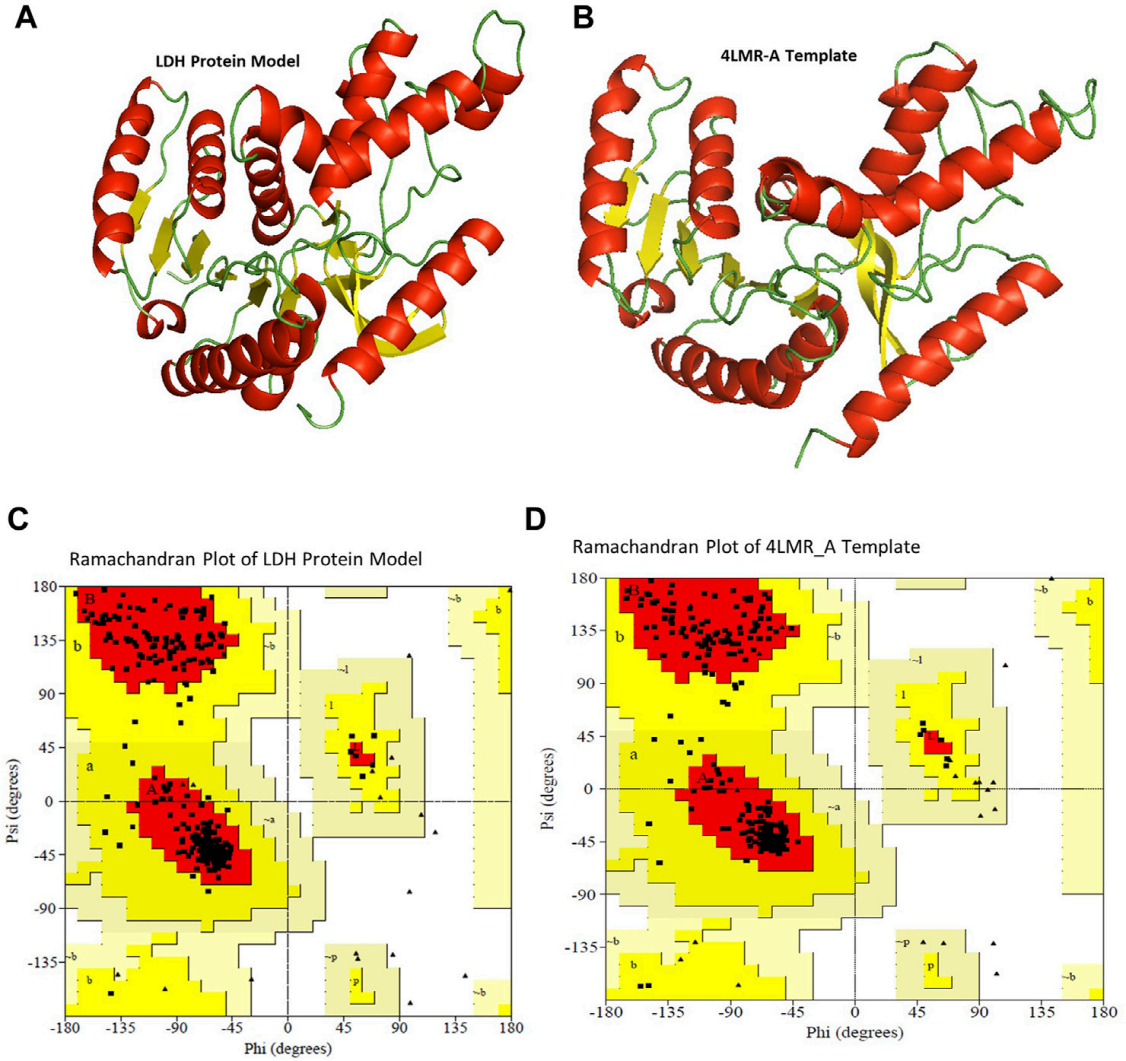
**(A, B)** Superimposition of model of LDH protein from *Bacillus cereus* (predicted model) and template *B. cereus* (PDB 4LMR_A) through cartoon representation. **(C, D)** Ramachandran plot for template *B. cereus* (PDB 4LMR_A) and model of *Bc*-LDH protein.

This study provides an insight into the molecular properties and the three-dimensional (3D) structure of the LDH protein expressed by the *B. cereus* strain NRC1. The 3D structure of the LDH protein expressed by the *B. cereus* strain NRC1 was evolved using the X-ray structure coordinate files of the dehydrogenase proteins from *B. cereus* (PDB 4LMR_A; identity 97.12%) (Figure 8B) and *B. stearothermophilus* (PDB ID 5LDN A; identity 62.26%) as templates. The alignment file, template file, and target file were provided by the PHYRE2 server, which was used to create the 3D structure of the *Bc*-LDH (Figure 8A). Using the Swiss PDB viewer’s YASARA Server force fields, the chosen model underwent energy minimization. These findings will be valuable for future comparative modeling and to further our understanding of the protein’s function.

### 3.9 Model assessment and validation of lactate dehydrogenase

The general stereochemical characteristics were used to evaluate the created model’s satisfaction. Using the Ramachandran plot, our 3D model of the LDH protein shows that 91.2, 8.8, 0, and 0% of the residues are in the most preferred regions, additional allowed regions, generously allowed regions, and disallowed regions, respectively, proving that the model is of high quality. The results were the same when the corresponding values for the template *B. cereus* (PDB 4LMR_A) were involved. A Ramachandran plot was constructed using the lactonase structures’ energy-minimized model. In the plot, the *x*-axis was divided into four quadrants: the forbidden area, the generously allowed region, the allowed region, and the low energy region (Supplementary Table S2S and Figures 8C,D). In addition, 94.22% was identified as the overall quality factor by both ERRAT and Verify 3D, and the compatibility between amino acid sequence (1D) and its model’s atomic model (3D) was 96.16. As can be seen in Supplementary Table S3, the Ramachandran plot indicates that the developed model is trustworthy and of high quality.

### 3.10 Docking and molecular interaction of 3D model of lactate dehydrogenase

**FIGURE 9:**
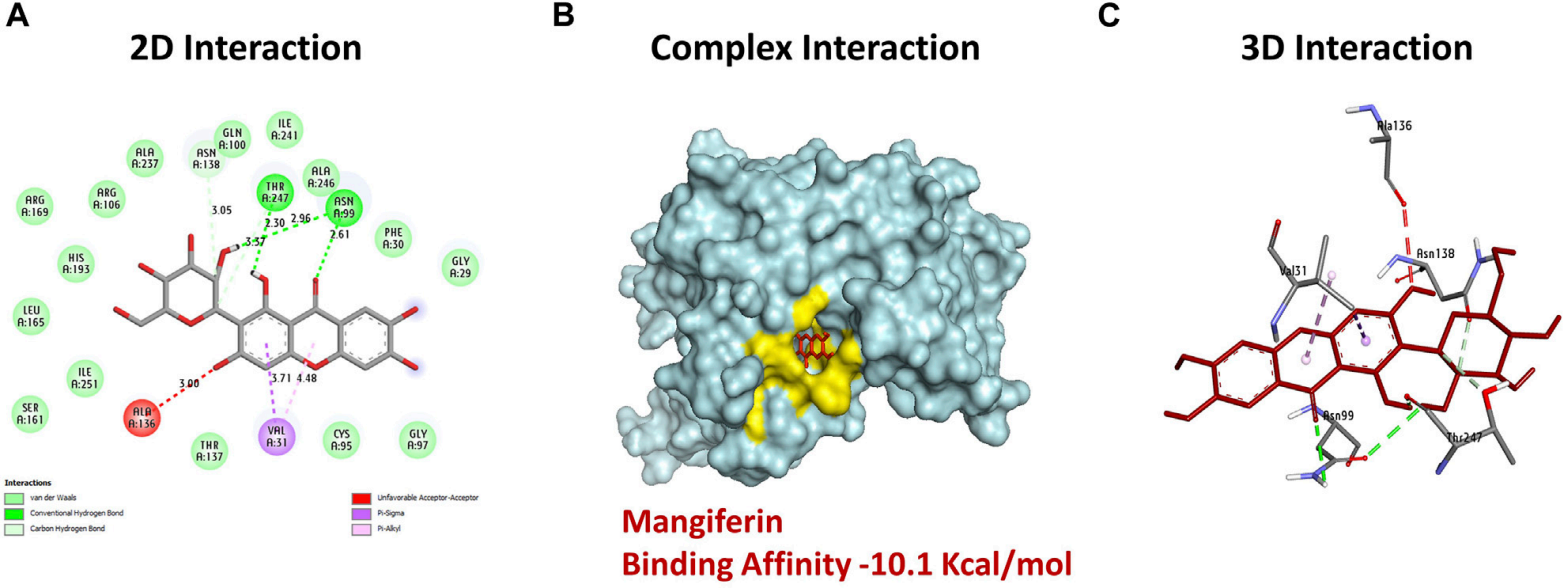
**(A)** 2D interaction: revealing hydrogen bond between ligands interacted with an amino acid (green dash line). **(B)** Complex interaction: showing binding of mangiferin with *Bc*-LDH displaying the most effective binding mode in the protein cavity (active site displayed by yellow color). **(C)** 3D interaction: showing the interacted amino acid residues (with ligand as color sticks).

Docking analysis of mangiferin, an inhibitor, to the 3D model of *Bc*-LDH’s binding energy with a focus on the interactions between mangiferin and the *Bc*-LDH protein was performed. The results of the docking analysis of the inhibitor mangiferin to the 3D model of *Bc*-LDH’s binding energy are shown in Supplementary Table S4. According to the results of the molecular docking, mangiferin had the most interactions with the 3D model of the *Bc*-LDH protein. In examining the docking results (Table 5S), Figures 9A–C shows that mangiferin, with an affinity value of −10.1 kcal/mol, shows docking with the best affinity interaction. Mangiferin is connected to Asn99, and Thr247 through three hydrogen bonds. Meanwhile, five non-hydrogen bond interactions were created in the activity pocket: Thr247 (carbon-H), Asn138 (Van der Waals), Ala136 (unfavorable bond), Val31 (pi–alkyl bond), and Val31 (pi–sigma bond). The amino acids involved in the ligand-3D model of *Bc*-LDH interactions are shown in bold red in the figure. This docking study demonstrates that the bonds formed by the amino acids Asn99 and Thr247 in the catalytic site improve the binding affinity. The 3D models of the *Bc*-LDH inhibitor mangiferin produced the highest results, with free binding energies of −10.1 kcal/mol when compared to the reference NADH. The results indicate that mangiferin could be a potential inhibitor for *Bc*-LDH, with the highest binding energy when compared to the reference NADH.

### 3.11 Molecular dynamic simulation

**FIGURE 10:**
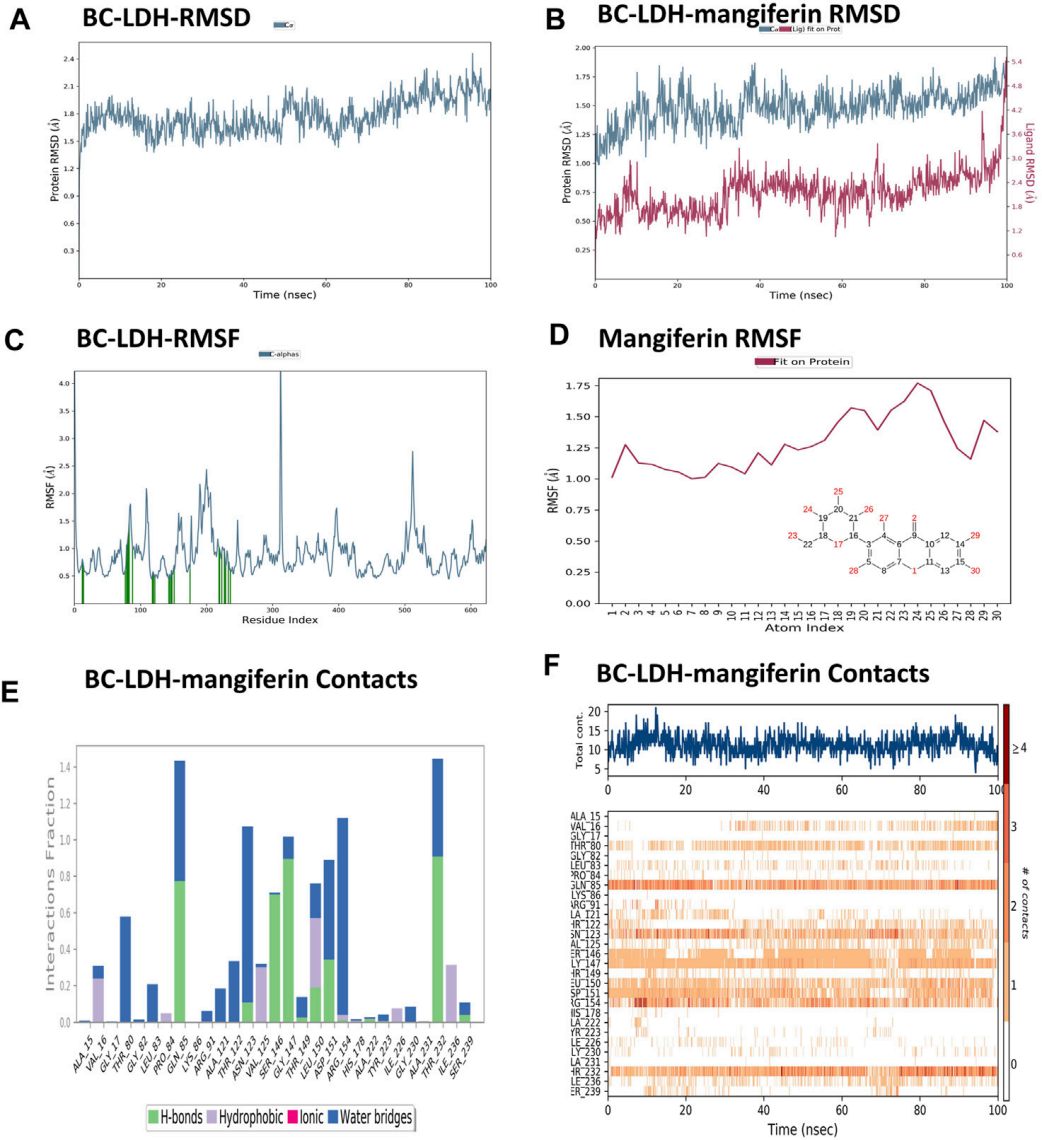
**(A)** RMSD plot of *Bc*-LDH protein, **(B)** RMSD plot of *Bc*-LDH-mangiferin complex, **(C)** RMSF plot of *Bc*-LDH protein, **(D)** RMSF plot of mangiferin, **(E, F)** *Bc*-LDH-mangiferin complex contact interactions.

MD simulation is the only technique used to investigate biomolecular interactions and has been utilized for the discovery of new inhibitors. Figure 10 depicts the MD simulation findings of *Bc*-LDH with mangiferin. Based on the RMSD plot analysis (Figure 10A), the backbone RMSD plot of the *Bc*-LDH system is equilibrated and stable at 1.8 Ǻ until 60 ns, after which it increases slightly to 2.1 Ǻ. For mangiferin (Figure 10B), the RMSD values increased by approximately 1.6 Ǻ, and the equilibrium was maintained at around 1.8 Ǻ after the procedure time of 35 ns, then increased slightly to 2.4 Ǻ from 40 ns through to the final of the simulation. On the other hand, the RMSD plot of the *Bc*-LDH-mangiferin complex structure attained a maximum RMSD value of approximately 1.6 Ǻ between 40 ns and 100 ns range. After comparison between the RMSD plot of LDH-mangiferin complex and *Bc*-LDH protein, we found that the *Bc*-LDH with mangiferin value was lower than the *Bc*-LDH protein's RMSD value, and all the systems found stability and equilibrium when their RMSD values fluctuated within a similar distance range of 1.0 Å and 1.6 Å. Furthermore, the RMSF was utilized to search how macromolecular proteins fluctuated locally at the residue level. Data obtained from Figure 10C show that the RMSF plot of *Bc*-LDH– mangiferin complex residues is less than 2.0 Ǻ, except for the residues having a maximum fluctuation in the region of 190th to 205th. In addition, the *Bc*-LDH residues engaged with mangiferin are denoted by green vertical bars. Also, mangiferin RMSF shows the ligand’s fluctuations broken down by atom, corresponding to the 2D structure in the top panel of Figure 10D. Additionally, throughout the simulation, interactions between *Bc*-LDH and mangiferin were monitored. These interactions can be classified into distinct types: hydrogen bonds, ionic interactions, hydrophobic interactions, and water bridges and be summarized as depicted in the plot shown in Figure 10E. Throughout the course of the trajectory, stacked bar charts are normalized. As shown in Figure 10F, the top panel displays the total number of individual connections made between *Bc*-LDH and mangiferin during the trajectory. In each trajectory frame, the bottom panel displays which residues interact with mangiferin. Some residues (Gln85, Gly147, and Thr232) have established many specific contacts with mangiferin, which are depicted by darker orange based on the scale given on the plot’s right.

**FIGURE 11:**
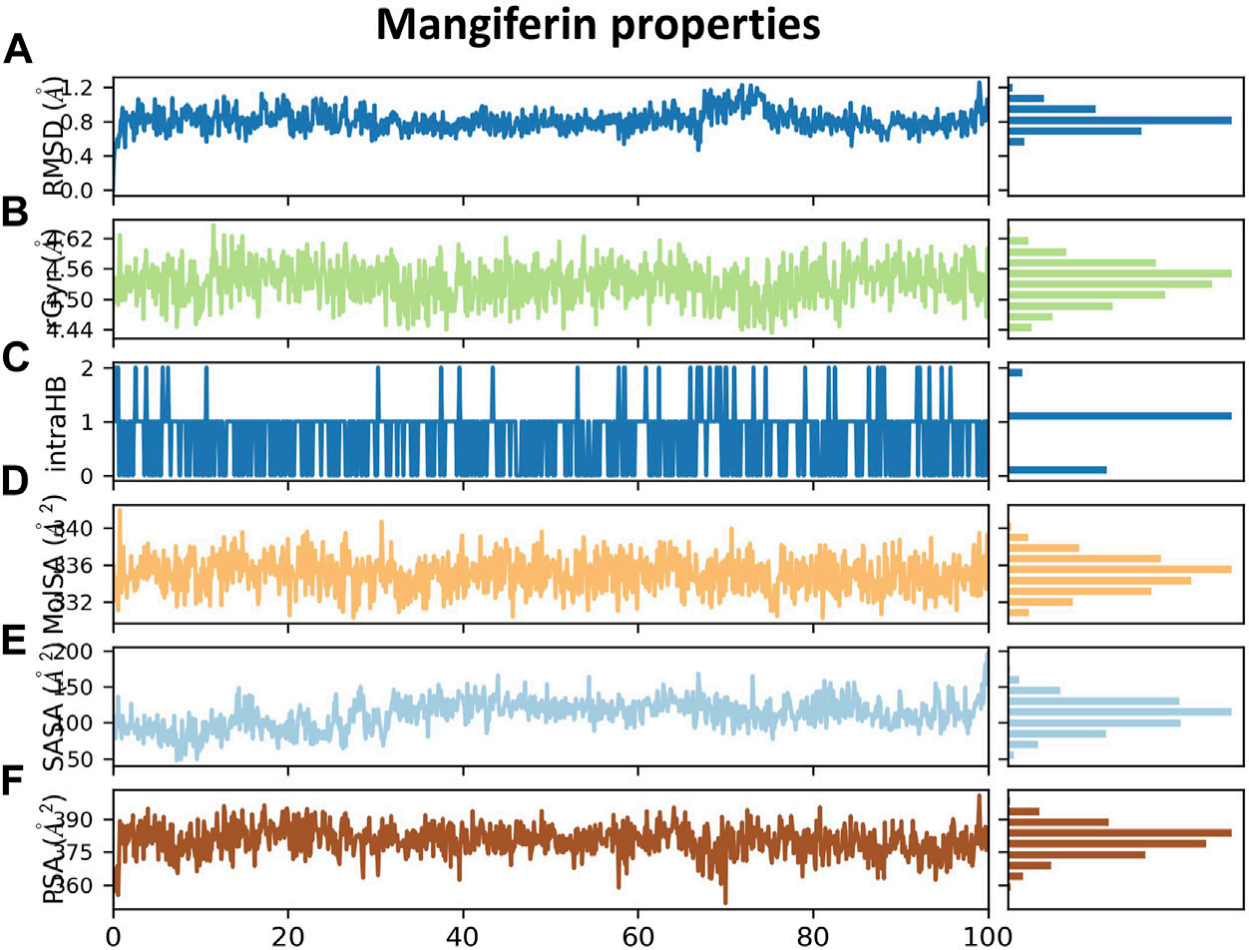
**(A)** RMSD plot of mangiferin, **(B)** rGyr of mangiferin, **(C)** intraHB within mangiferin molecule, **(D)** MolSA of mangiferin, **(E)** SASA of mangiferin, and **(F)** PSA of mangiferin.

Data obtained from Figure 11A show different properties of mangiferin such as the RMSD of a ligand with respect to the reference conformation, with typically the first frame being used as the reference and regarded such that time t = 0 and its fluctuations are between 0.6–0.8 Å. Also, Figure 11B shows that the rGyr of mangiferin is between 4.50 and 4.55 Å, which measures the ‘extendedness’ and is equivalent to its principal moment of inertia. Furthermore, Figure 11C indicates a number of internal hydrogen bonds “Intramolecular Hydrogen Bonds (intraHB)” within the mangiferin molecule. In addition, the molecular surface area (MolSA), solvent accessible surface area (SASA), and polar surface area (PSA) were calculated for mangiferin as obtained from Figures 11D–F, respectively.

### 3.12 Inhibition by mangiferin

**FIGURE 12:**
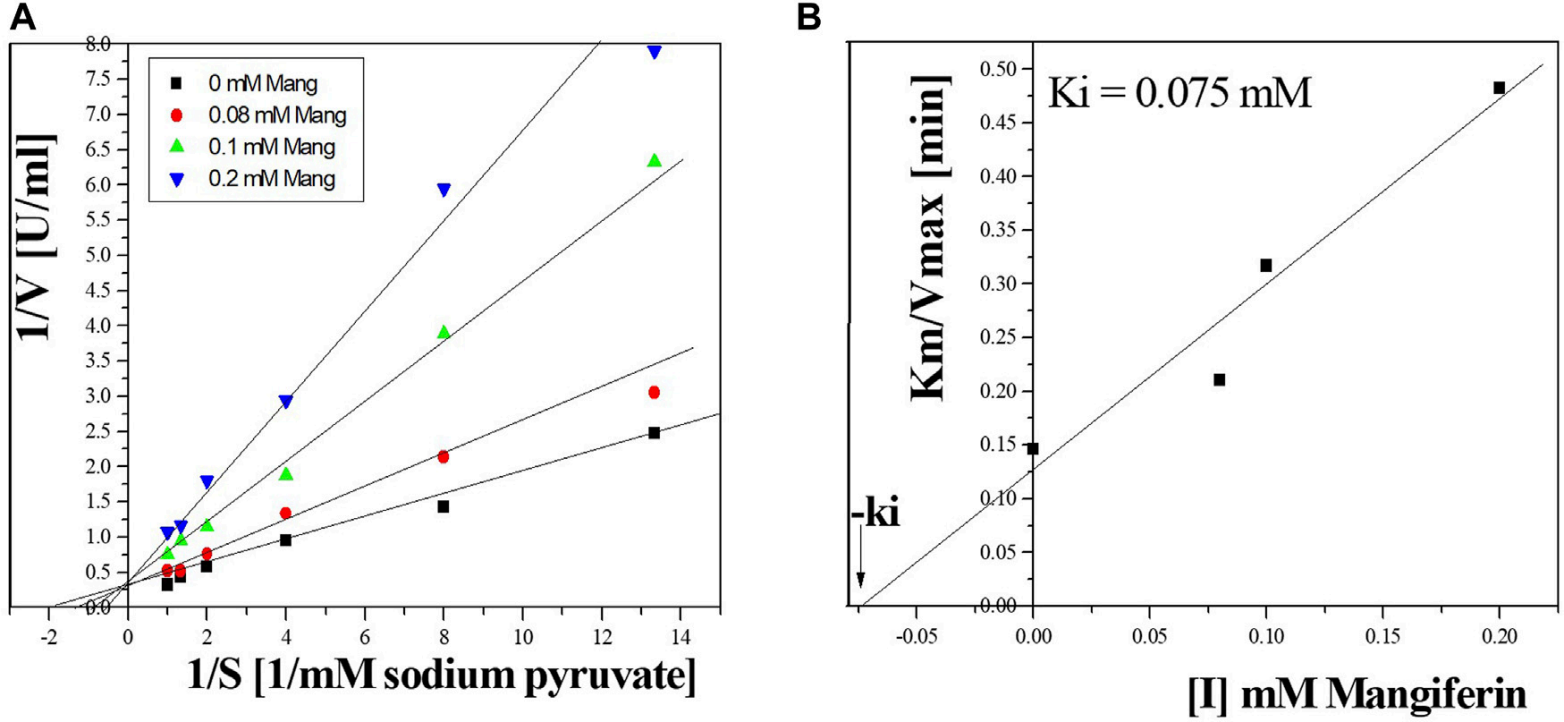
**(A)** Lineweaver-Burk plots reveal the LDH inhibition type using mangiferin. **(B)** Estimation of the inhibition constant (Ki) for LDH inhibition using mangiferin.

The effect of mangiferin on *Bc*-LDH activities was estimated. The physiochemical parameters of various compounds, such as mangiferin, lactic acid, pyruvate, and NADH, are shown as the *Bc*-LDH active site effectors in Supplementary Table S4, where mangiferin inhibited the purified enzyme vigorously. The activity of *Bc*-LDH was decreased by increasing mangiferin (Supplementary Table S4). Figures 12A, B reveals that mangiferin competitively inhibited *Bc*-LDH activity with the ki of 0.075 mM.

## 4 Discussion

Ten bacterial isolates were successfully isolated from the Egyptian soil. From which, only one isolate could outgrow itself and exhibit the highest LDH activity. Using 16s rDNA sequencing, this NRC1 isolate was subjected to molecular analysis. As shown in Figure 1, the universal primers successfully amplified a 1,500-bp DNA fragment that corresponds to the 16s rDNA fragment. The 16s rDNA fragment’s nucleotide sequences were compared to every piece of information in the GenBank database. According to the aligned results, the bacterial isolate NRC1 shared 98.97% of its DNA homology with *B. cereus*. The tested NRC1 had a close taxonomic link to the *Bacillus* genus, according to the phylogenetic research. The selected strain was identified as *B. cereus* NRC1, submitted to the GenBank database, and given the accession number LC706200.1*.* The *B. cereus* strain NRC1 was grouped closely with the *B. cereus* strain ATCC (with the accession no. Q81EP4) with 99.04% sequence similarity. This outcome was comparable to that of El-Sayed et al. (2019). They used 16s rDNA sequencing to analyze a strain of *Lysinibacillus sphaericus* that produces *ß*-glucosidase isolated from an Egyptian environment. Also, Abosereh et al. (2022) used 16s rDNA barcoding for molecular identification of a *Bacillus* sp. isolated from the Egyptian soil and identified it as *B. cereus*. Furthermore, other researchers have achieved comparable outcomes by isolating LDH-producing *B. cereus*, and enhancing its expression and purification yields in *E. coli*, thereby making *E. coli* a better candidate for LDH production (Andreeßen et al., 2018).

In our study, we successfully amplified the ∼945-bp *Bc*-LDH gene from the *B. cereus* NRC1 strain and cloned it in the pTZ57R/T vector, then transformed it to the *E. coli* DH5α™ after cloning in the pTZ57R/T vector, and the clone was given the name LDH-pTZ57R/T. Following PCR confirmation of the insert in the clone, the sequence was placed in the Genebank as LC706200.1. Additionally, the LDH gene was cloned into the pET-28a (+) vector, yielding the LDH-pET clone, whose expression was triggered by 0.1 mM IPTG. These results corroborate with Goto et al.’s (2018) findings of cloning, sequencing, and identifying the LDH gene by heterologous expression in *E. coli* DH5α™. Additionally, with the help of the plasmid pET28a, the Ruminococcaceae strain CPB6 gene encoding L-LDH was effectively cloned and expressed in *E. coli* BL21 (DE3) (Yang et al., 2020). Subsequently, the bioinformatics analysis of the NRC1-LDH protein classifies *Bc*-LDH-NRC1 among the known L-LDH protein family domain. Also, all 11 curated LDH protein sequences were retrieved from the UniProt database, drawn from the database, and matched to diverse organisms for phylogenetic analyses. The data set contained *Bacillus* LDH proteins. The results of the comparison research showed that LDH shared 99% of its sequence with homologous LDH proteins. Besides, six amino acids represented the LDH’s active site (175–181). Specifically, amino acid His178 serves as the catalytic center of the active site for substrate binding. The SignalP 6.0 server then verified signal peptides and their cleavage sites, and the outcomes contained residues ranging from 1 to 17. These results are similar to those of Yang et al. (2020). Also, similar results were obtained by Andreeßen, et al. (2018) who reported that they clone and express the LDH gene from *B. cereus* in *E. coli* DE3, resulting in successful overexpression of LDH from *B. cereus* in the soluble cell fraction. However, they found that LDH from both *B. cereus* and *E. coli* was primarily detectable in the crude extract. In addition, Williams et al. (2022) reported that a d-lactate dehydrogenase (D-LDH) gene was isolated, immobilized, and purified from *Lactobacillus helveticus.* Also, a NADH-dependent D-lactate dehydrogenase gene was cloned, engineered for changing the substrate specificity, and homology modeled from *Lactobacillus fermentum* (Fan et al., 2021).

LDH from *B. cereus* underwent three purification steps. The purified *Bc*-LDH in a purification fold of 25.5 showed a specific activity of 22.7 units/mg protein. The findings were close to those of Helmy et al. (2019) reported, indicating that the purified LDH enzyme obtained from buffalo liver displayed a specific activity of 17.6 units/mg protein, with purification folds of 16. Additionally, LDH from pig heart exhibited a specific activity of 26.93 U/mg protein and 54.96 purification fold (Karamanos, 2014), and yak LDH-A where the specific activity of 103 units/mg protein and 18.7 purification fold (Yucai et al., 2008) and purified LDH of *Lactobacillus* sp. SK007 also results in specific activities of 203.30 U/mg and 34.29 purification fold (Li et al., 2008). By contrast, LDH isolated from the male chicken from Ebocha oil yielded specific activities of 9,090.28 U/mg and 2.25 purification fold (Esonu et al., 2019), and LDH from *Plasmodium knowlesi* showed specific activities of 475.6 U/mg and 1.6 purification fold (Salim et al., 2021).

The molecular weight estimated by using the gel filtration calibration curve (194.5 kDa) was approximately fourfold as it was determined by SDS-PAGE (35.0 kDa). These results suggest that the *Bc*-LDH enzyme is a tetramer in solutions and appears as a monomer on SDS-PAGE. Similar results have been recorded by Savijoki and Palva (1997) for L-lactate dehydrogenase (ldhL) of *L. helveticus*.

The pH stability and optimum pH were established. The pure *Bc*-LDH from *B. cereus* has the maximum specific activity, according to optimal pH values, at pH 8.0 using Tris-HCl buffer. The *Bc*-LDH was stable at pH ranging from 5.5 to 9.0 (Figure 6). This is closely connected to the recombinant LDH of the parasite *Theileria annulata* and similar to the findings in bacterium *L. fermentum* (Chen et al., 2017) and wild-type TaLDH (Nural et al., 2016). Additionally, the pH optima herein is close to the pH optima of LDH from psychrophilic marine bacterium and the LDH-2 of the shrimp (Fregoso-Peñuñuri et al., 2017), LDH-A of the Antarctic krill *Euphausia superba* (Mulkiewicz et al., 2001) and LDH from the liver of the lizard Varanus (Javed et al., 1997). LDH extracted from the pig heart of *G. domesticus* interestingly revealed an ideal pH of 6.0 (Esonu et al., 2019).

At 2 mM, ZnCl_2_ and CuCl_2_ reduced *Bc*-LDH activity by 20% and 30%, respectively. However, at 5 mM of both cations, ZnCl_2_ lowered *Bc*-LDH catalytic power by 40% while CuCl_2_ lowered it by 48%. Nevertheless, the activity of *Bc*-LDH was greatly reduced at high zinc concentrations as observed in contaminated environmental conditions, despite physiological amounts of copper and zinc not affecting the enzyme activity. These findings are comparable to those obtained with the white shrimp *Litopenaeus vannamei* LDH enzyme. These findings are consistent with human erythrocytes LDH, which show that adding magnesium would increase the enzymatic activity (Leyva-Carrillo et al., 2019).

Next, based on the *in silico* prediction of the secondary structure for *Bc*-LDH protein and according to the high similarity and homology between the LDH protein structures, BLAST against PDB demonstrated that the derived sequence of *Bc*-LDH-NRC1 demonstrated the strongest identity 97.12% with *B. cereus* (PDB 4LMR_A). So, using the 3D structure and homology modeling of LDH made from *B. cereus* NRC1, the 3D structure of the *Bc*-LDH-NRC1 protein was created. The online Ramachandran plot produced by the PROCHECK server determined that the 3D model of *Bc*-LDH-NRC1 was of greater quality than other models. In our *in silico* simulation, by using binding affinity and molecular interaction in molecular docking, a number of putative inhibitory interacting molecules were assessed.

The dynamic trajectory output data sets the ground for determining the relationship between the structure and function of proteins (Dey et al., 2021). The complex structure of the LDH and mangiferin was further evaluated using 100 ns molecular dynamics simulation using Desmond simulation software of Schrödinger LLC. The complex motion can be reflected by the RMSD obtained from the molecular dynamics simulations. The RMSD plots of themangiferin and LDH-mangiferin complex indicates that all systems experienced an initial kinetic shock, leading to a rapid increase in the RMSD values. Following this, the RMSD readings of the systems fluctuated between a consistent distance indicating that the systems had achieved stability and equilibrium. The RMSF of the LDH-mangiferin system was determined to explore the conformational behavior of proteins following binding to small molecule ligands. The RMSF values of each residue in the LDH complex were found to be within the range of 0.5–1.5 Å, suggesting that the compound did not significantly affect the protein’s stability. Also, Figure 11C depicts the findings that the LDH-mangiferin system demonstrated an elevated number of hydrogen bonds, indicating that the support from and affinity of combinations were strong. Also, more hydrogen bonding interactions for greater stability of the mangiferin with the LDH protein were generated. These results are similar and in agreement with those of Dhal et al. (2018), who reported *in silico* screening of small molecule inhibitors against LDH of *Cryptosporidium parvum*. Mahapatra and Das (2020) observed similar results after performing *in silico* docking, molecular dynamics modeling, and the binding of gentianine to the LDH, which acts as a crucial enzyme for the parasite’s survival.

Our results have shown that mangiferin exhibits a great change in the binding free energy for *Bc*-LDH-NRC1. Also, we have discovered that mangiferin is promising as an inhibitor. These results are similar and in agreement with those of Dhal et al. (2018) and Sun et al. (2015), who performed molecular docking to screen for novel inhibitors of LDH enzymes.

Mangiferin promotes apoptosis in tumor cells and inhibits the cell cycle (Du et al., 2018). Thus, based on these results, polyphenols such as mangiferin are potential candidates for the development of chemotherapeutic drugs by restricting LDH.

## 5 Conclusion

Our study describes the isolation, sequencing and cloning, *E. coli* expression, and biochemical characterization of *Bc*-LDH from a novel *B. cereus* NRC1 species. The recombinant enzyme’s 3D model was created, verified, and *in silico* matched with the template LDH protein. We rated many inhibitors by estimating the binding free energy changes with various protein partners, highlighting the conserved structural and functional domains, such as the active site residues. The low average RMSF revealed that individual amino acid residues were stable in the protein’s dynamic state during the MD simulation. Findings for the selection and use of inhibitor compounds for LDH activity can be extended to plant-based bioactive anticancer drugs.

## Data Availability

The data sets presented in this study can be found in online repositories. The names of the repository/repositories and accession number(s) can be found in the article/Supplementary Material.

## Abbreviations

*Bc*-LDH, *Bacillus cereus*–lactate dehydrogenase; BL21(DE3), *Escherichia coli* BL21(DE3); DTT, dithiotheritol; PMSF, phenylmethylsulfonyl fluoride; and Ki, inhibition constant.
