# Supplementary material for: Recombinant production, purification, and biochemical characterization of a novel L-lactate dehydrogenase from Bacillus cereus NRC1 and inhibition study of mangiferin
Source: Front Bioeng Biotechnol. 2023 Apr 6;11:1165465. doi: 10.3389/fbioe.2023.1165465 (PMC10117910; doi:10.3389/fbioe.2023.1165465)
Supplement: Supplementary file 1 [file DataSheet1.docx]

**Table 1S**

**The implication of divalent cations on the *Bc-*LDH.**

| **Reagent** | **Concentration**  (mM) | **LDH Residual activity**  (%) |
| --- | --- | --- |
| **Control** | ----- | 100±0.0 |
| **CoCl_2_** | 2.0 | 100±8.2 |
|  | 5.0 | 120±15.3 |
| **MnCl_2_** | 2.0 | 95±6.4 |
|  | 50 | 90±9.4 |
| **FeCl_2_** | 2.0 | 64±11.2 |
|  | 5.0 | 35±6.1 |
| **ZnCl_2_** | 2.0 | 80±9.2 |
|  | 5.0 | 60±4.3 |
| **CuCl_2_** | 2.0 | 70±8.5 |
|  | 5.0 | 52±6.6 |
| **NiCl_2_** | 2.0 | 90±10.7 |
|  | 5.0 | 79±7.2 |
| **MgCl_2_** | 2.0 | 100±12.6 |
|  | 5.0 | 103±14.4 |
| **CaCl_2_** | 2.0 | 90±8.7 |
|  | 5.0 | 81±9.5 |

**Table 2S**

Plot statistics of template and generated model of Ramachandran plot.

| Area | Plot statistics of template | | Plot statistics of model | |
| --- | --- | --- | --- | --- |
|  | Number of residues | % | Number of residues | % |
| Residues in most favored regions [A, B, L] | 249 | 91.2 | 250 | 91.2 |
| Residues in additional allowed regions [a, b, l, p] | 24 | 8.8 | 24 | 8.8 |
| Residues in generously allowed regions [~a, ~b, ~l, ~p] | 0 | 0.0 | 0 | 0.0 |
| Residues in disallowed regions | 0 | 0.0 | 0 | 0.0 |
| Number of non-glycine and non-proline residues | 273 | 100 | 274 | 100 |
| Number of end-residues (excl. Gly and Pro) | 2 | | 2 | |
| Number of glycine residues (shown as triangles) | 26 | | 26 | |
| Number of proline residues | 11 | | 11 | |
| Total number of residues | 312 | | 313 | |

**Table 3S**

Evaluation plot statistics results of model and template.

| Protein | **PROCHECK** | | | | **Verify 3D** | **ERRAT** |
| --- | --- | --- | --- | --- | --- | --- |
|  | **Most favored**  **regions** | **Additional Allowed**  **regions** | **Generally allowed**  **regions** | **Disallowed regions** | **3D-1D**  **Score** | **Quality Factor** |
| **Model** | **91.2%** | **8.8%** | **0.0%** | **0.0%** | **94.22%** | **96.16** |
| **Template** | **91.2%** | **8.8%** | **0.0** | **0.0** | **94.77%** | **97.98** |

**Table 4S**

The Physiochemical parameters of mangiferin, lactic acid, pyruvate and NADH screened for Lipinski’s rule and comparative binding affinity with 3D model of lactate dehydrogenase.

| **Ligand name** | **PubChem id** | **Molecular Weight** | **Binding Affinity** | **Hydrogen**  **Bond Donor** | **Hydrogen Bond**  **Acceptor** | **Rotatable**  **bonds** | **logP** |
| --- | --- | --- | --- | --- | --- | --- | --- |
| Lactic acid | 612 | 90.08 | -3.7 | 2 | 3 | 1 | -0.7 |
| Mangiferin | 5281647 | 422.3 | -10.1 | 8 | 11 | 2 | -0.4 |
| Pyruvate | 107735 | 87.05 | -3.9 | 0 | 3 | 0 | -0.6 |
| NADH (Ref.) | 439153 | 665.4 | -10.3 | 8 | 19 | 11 | -5.7 |

**Table 5S**

Molecular interactions of amino acids restudies; of the 3D model of lactate dehydrogenase, with various ligands (amino acids showing similar interactions are marked in bold and red color).

| No. | 3D model of lactate dehydrogenase **Protein** | **Ligand** | **3D Structure** | **Hydrophilic Interactions** | | **Hydrophobic Contacts** | | **No. of**  **H-Bonds** | **No. of**  **Total Bonds** |
| --- | --- | --- | --- | --- | --- | --- | --- | --- | --- |
|  |  |  |  | **Residue (H- Bond)** | **Length** | **Residue (Bond type)** | **Length** |  |  |
| 1 |  | Lactic acid | 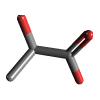 | Gln100, (H- Bond) | 2.14 | Asn99, (Van der Waals) | 3.43 | 1 | 2 |
| 2 |  | Mangiferin | 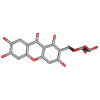 | Asn99, (H- Bond)  Asn99, (H- Bond)  **Thr247, (H- Bond)** | 2.61  2.96  2.30 | Val31, (pi-alkyl bond)  Val31, ((pi-sigma bond)  Ala136, (Unfavorable bond)  Thr247, (carbon-H)  Asn138, (Van der Waals) | 4.48  3.71  3.00  3.37  3.05 | 3 | 8 |
| 3 |  | Pyruvate | 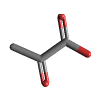 | Thr247, (H- Bond)  Arg106, (H- Bond)  Arg169, (H- Bond)  His193, (H- Bond) | 2.00  2.49  2.30  2.33 | - |  | 4 | 4 |
| 4 |  | NADH | 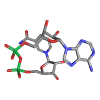 | **Val31, (H- Bond)**  Gly29, (H- Bond)  Gly32, (H- Bond)  Phe30, (H- Bond)  Asn99, (H- Bond)  Asp52, (H- Bond)  Ala136, (H- Bond)  **Thr247, (H- Bond)** | 2.25  2.17  2.21  2.12  2.38  4.95  2.18  2.98 | Ala98, (Unfavorable bond)  Gly97, (carbon-H)  Ala96, (alkyl bond)  Ala53, (alkyl bond)  Ile116, (alkyl bond) | 2.89  3.51  3.99  4.38  4.84 | 8 | 13 |

**Table 6S**

Effect of mangiferin as inhibitor for lactate dehydrogenase.

| **Compounds** | **Concentration**  (mM) | **Inhibition%** |
| --- | --- | --- |
| **Mangifrein** | 0.08 | 25 |
|  | 0.1 | 58 |
|  | 0.2 | 68 |
|  | 0.4 | 77 |
|  | 0.08 | 93 |
|  | 1.0 | 95 |


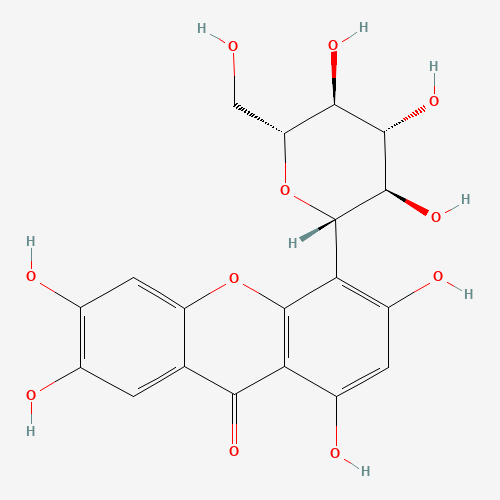


**Fig. 1S**: Structure of mangiferin


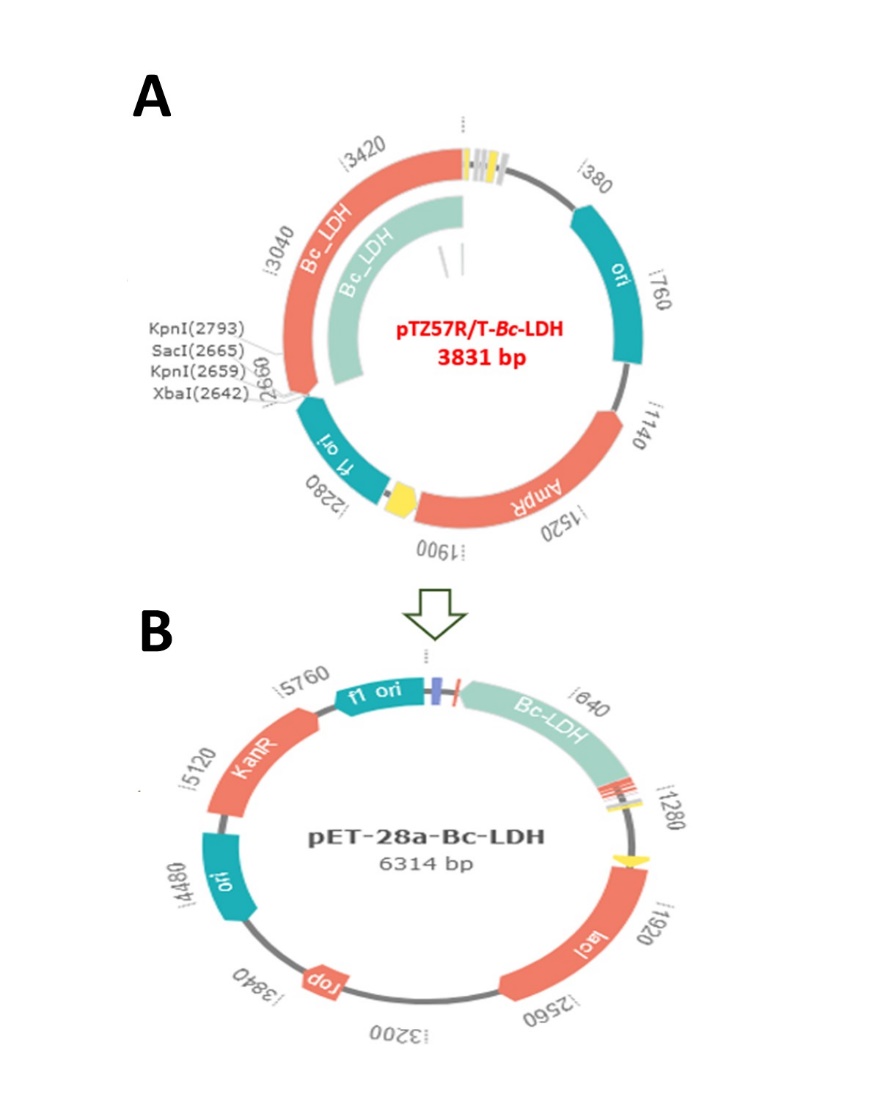


**Fig. 2S**: . **(A)** Schematic representation of pTZ57R/T vector carrying *Bc*-LDH. **(B)** Created recombinant plasmid pET28- *Bc*-LDH.


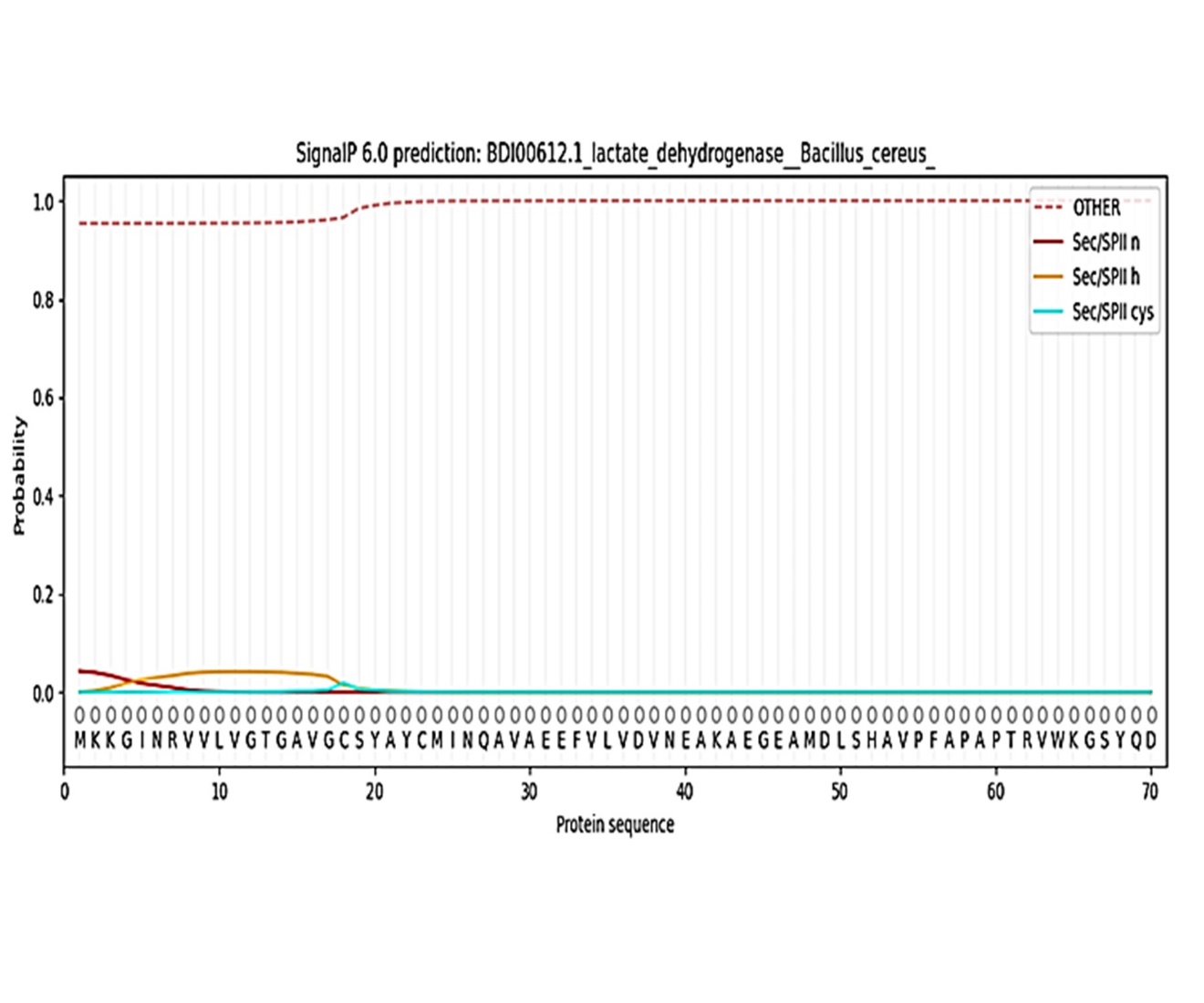


**Fig. S3**: Signal IP prediction of amino acids for *Bc*-LDH gene from *B. cereus* NRC1.


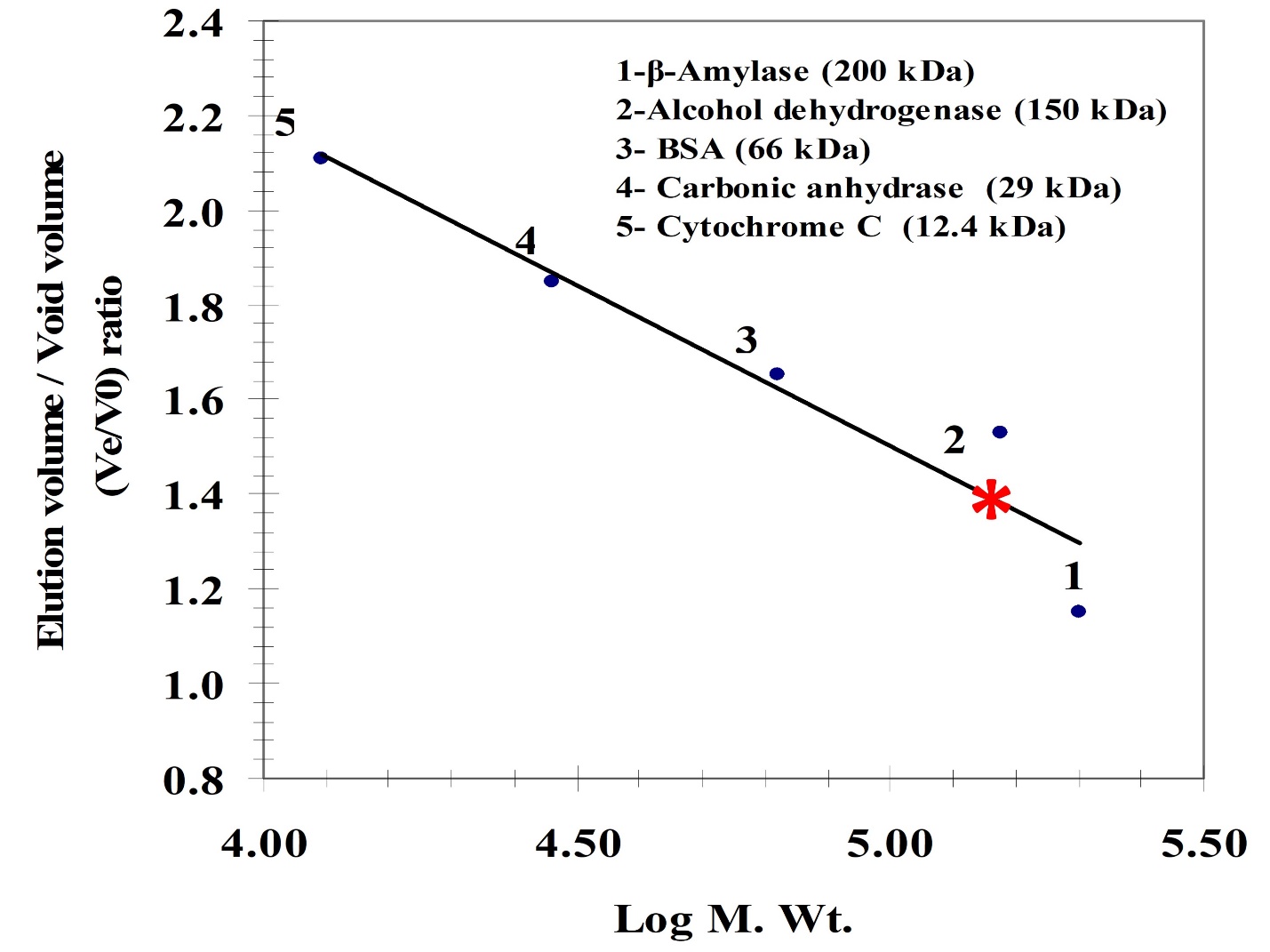


**Fig. S4**: A calibration curve constructed by gel filtration on the Sephacryl S-300 column for the molecular weight determination of *Bc*-LDH. (_*_) denotes *Bc*-LDH calibrated molecular weight.
